# Characterization of a Gene Expression Signature in Normal Rat Prostate Tissue Induced by the Presence of a Tumor Elsewhere in the Organ

**DOI:** 10.1371/journal.pone.0130076

**Published:** 2015-06-15

**Authors:** Hanibal Hani Adamo, Sofia Halin Bergström, Anders Bergh

**Affiliations:** Department of Medical Biosciences, Pathology, Umeå University, Umeå, Sweden; King's College London, UNITED KINGDOM

## Abstract

Implantation of rat prostate cancer cells into the normal rat prostate results in tumor-stimulating changes in the tumor-bearing organ, for example growth of the vasculature, an altered extracellular matrix, and influx of inflammatory cells. To investigate this response further, we compared prostate morphology and the gene expression profile of tumor-bearing normal rat prostate tissue (termed tumor-instructed/indicating normal tissue (TINT)) with that of prostate tissue from controls. Dunning rat AT-1 prostate cancer cells were injected into rat prostate and tumors were established after 10 days. As controls we used intact animals, animals injected with heat-killed AT-1 cells or cell culture medium. None of the controls showed morphological TINT-changes. A rat Illumina whole-genome expression array was used to analyze gene expression in AT-1 tumors, TINT, and in medium injected prostate tissue. We identified 423 upregulated genes and 38 downregulated genes (p<0.05, ≥2-fold change) in TINT relative to controls. Quantitative RT-PCR analysis verified key TINT-changes, and they were not detected in controls. Expression of some genes was changed in a manner similar to that in the tumor, whereas other changes were exclusive to TINT. Ontological analysis using GeneGo software showed that the TINT gene expression profile was coupled to processes such as inflammation, immune response, and wounding. Many of the genes whose expression is altered in TINT have well-established roles in tumor biology, and the present findings indicate that they may also function by adapting the surrounding tumor-bearing organ to the needs of the tumor. Even though a minor tumor cell contamination in TINT samples cannot be ruled out, our data suggest that there are tumor-induced changes in gene expression in the normal tumor-bearing organ which can probably not be explained by tumor cell contamination. It is important to validate these changes further, as they could hypothetically serve as novel diagnostic and prognostic markers of prostate cancer.

## Introduction

Prostate cancer, a very common multifocal disease with highly variable behavior, is difficult to diagnose and prognosticate [1, 2]. The diagnosis is dependent on microscopic examination of needle biopsies of prostate tissue. Unfortunately, current imaging techniques cannot identify prostate cancers and safely guide biopsy needles towards tumors. The current way of overcoming this problem is to take multiple biopsies, but as biopsies only sample a minute part of the whole prostate they can miss all the tumor tissue present, or the most malignant tissue. Globally, of the millions of men who have prostate examinations every year due to the suspicion of cancer, most have biopsies that are negative for cancer [2]. Whether this indicates that cancer is not present at all or has been missed is not generally known, but in 20% of the men examined, cancer is detected in a subsequent round of biopsies [2]. If, however, the benign tissue sampled in prostate biopsies is in some way altered by the presence and nature of tumors elsewhere in the organ, this could possibly lead to an improvement in diagnosis.

In order to grow and spread, tumors need to interact with adjacent and more remote tissues [3, 4]. Tumor cells, for example, interact with the closely adjacent tumor stroma, and also with distant organs such as the bone marrow and pre-metastatic niches [3, 5]. One additional—often neglected—site that is likely to be affected both by the needs of the growing tumor and the host defense is the tumor-bearing organ. We have therefore hypothesized that aggressive cancers affect the tumor-bearing organ in ways that are quantitatively and qualitatively different from the effects of more indolent tumors, and that a better understanding of this might lead to better diagnosis and treatment of prostate cancer [1].

We have shown that implantation of a rat prostate tumor induces tumor-stimulating morphological changes in the surrounding normal rat prostate tissue. The density of inflammatory cells, such as macrophages and mast cells, is increased and facilitates both growth of the feeding vasculature and tissue remodeling [1, 6–8]. The extracellular matrix is altered; expression of hyaluronan, for example, is increased and this promotes tumor growth [9]. Although appearing morphologically unaffected, luminal glandular epithelial cells show a delayed apoptosis response to castration [6]. Similarly, in patients, alterations in the epithelium and stroma of the non-malignant prostate tissue surrounding tumors are related to prognostically important tumor characteristics such as Gleason score and tumor stage, and can be used to evaluate the risk of death from prostate cancer in a watchful waiting cohort [1, 8–14]. We have suggested a novel term for this type of normal tissue affected by the presence of an adjacent tumor: tumor-instructed (and thus indicating) normal tissue (TINT) [1]. TINT contains both morphologically normal-appearing epithelium and stroma of the tumor-bearing organ and is not in direct contact with the tumor epithelial cells; it is thus different from the already well-established tumor stroma.

To explore the TINT concept in more detail we now investigated if the gene expression pattern in TINT was different from that in non tumor-bearing prostates. We compared the prostate tissue surrounding the poorly differentiated and locally aggressive Dunning AT-1 prostate tumor with that in prostate tissue from tumor-free animals and found 461 genes whose expression was altered (423 up-regulated and 38 down-regulated) in TINT compared to controls. This suggested that the presence of a tumor altered gene expression in the tumor-bearing organ. The TINT gene signature was linked to processes such as extracellular matrix organization, immune responses, and inflammation. Alterations in gene expression in TINT suggest that tumors exploit the surrounding tumor-bearing organ for their own benefit, and such changes could therefore serve as potential therapeutic targets and/or as diagnostic/prognostic markers for prostate cancer.

## Materials and Methods

### Cells and animals

The fast-growing, androgen-insensitive, anaplastic and low-metastatic Dunning rat AT-1 prostate tumor cells (ECACC; Sigma Aldrich, Stockholm, Sweden) were grown in culture as described earlier [15]. For implantation of the AT-1 tumor cells, adult male Copenhagen rats were used (Charles River, Sulzfeld, Germany) [6]. All the animal work was approved by the Umeå ethical committee for animal research (Permit Number: A110-12) and strong efforts were made to minimize animal discomfort and suffering.

### Animals for morphological analyses

To study tissue morphology and to label hypoxic and proliferating cells, animals were anesthetized and injected with 2000 AT-1 cells in 40 μl RPMI-1640 cell culture medium into one lobe of the ventral prostate (VP) using a Hamilton syringe (*n* = 7). Control animals were injected with heat killed AT-1 cells (100°C for 30 minutes) in RPMI (*n* = 8) or with RPMI medium alone (*n* = 9). At day 10 (when the tumors occupied about 60% of the prostate volume, see below), the animals were injected with Hypoxyprobe (Millipore, Bedford, MA) and bromodeoxyuridine, BrdU (Roche, Mannheim, Germany) one hour before sacrifice, as previously described [6], and the prostates, livers, and kidneys were formalin-fixed and embedded in paraffin. Paraffin-embedded tissues were sectioned and stained with an antibody to Hypoxyprobe (NPI, Inc. Burlington, MA, US), and the percentage of hypoxic prostate tissue was determined as previously described [6]. Sections were also stained with antibodies against factor VIII (Dako, Denmark), CD68 (Serotec, Oxford, UK) and BrdU (BD Biosciences, CA, US) and with toluidine blue to label blood vessels, macrophages, proliferating cells and mast cells, respectively, and to determine the volume density of these tissue components and the endothelial BrdU labelling index using methods earlier described [6–8]. The Mann-Whitney U test was used for comparisons between groups and any *p*-value *<* 0.05 was considered significant.

### Animals for gene expression analyses

For the microarray studies, animals were implanted with AT-1 cells (*n* = 11) or RPMI as controls (*n* = 15) as described above. The animals were sacrificed at day 10 and the prostate lobes were quickly removed, frozen in liquid nitrogen, and stored at -80°C.

For the RT-PCR, a new set of animals with AT-1 tumors (*n* = 8), RPMI only (*n* = 10) or with heat-killed AT-1 cells (*n* = 8) was used.

Five-μm thick cryostat sections of the VP lobe were taken for pathological evaluation, in order determine the size and location of the tumor and the surrounding non-malignant prostate tissue in the samples, and verify that the VP lobe from control animals was free of tumors and other pathologies. Surrounding non-malignant prostate tissue and prostate tumor tissue were dissected with a margin of 0.5 to 1 mm to avoid contamination from each other (S1 Fig). When sufficient tissue had been collected an additional cryo-section was cut to verify that the tissue dissected contained only the intended tissue type.

### RNA extraction

Total RNA from 35 samples (8 tumors, 11 TINT, 15 normal prostate controls, and one batch of AT-1 cells) was extracted using TRIzol according to the manufacturer’s instructions (Invitrogen, Stockholm, Sweden). The concentration of total RNA from each sample was measured with a Nanodrop ND-1000 spectrophotometer (Nanodrop Technologies, Wilmington, DE). The integrity of the RNA was determined using an Agilent 2100 BioAnalyzer (Agilent, Willmington, DE).

#### Preparation of cRNA and Illumina chip hybridization

Biotin-labeled cRNA was synthesized from 200 ng total RNA using the IlluminaTotalPrep RNA Amplification kit (Applied Biosystems, Austin, TX) according to the manufacturer’s protocol. The quality of labeled cRNA was verified using a Nanodrop ND-1000 spectrophotometer. A total of 750 ng biotin-labeled cRNA from each sample was loaded onto the 12-sample RatRef Illumina BeadChip gene expression array (Illumina, San Diego, CA) according to the manufacturer’s protocols. The arrays were scanned and fluorescence signals measured using the Illumina BeadArray Reader (Illumina, San Diego, CA, USA).

### Data analysis

The array data were analyzed with GenomeStudio software (version 2009.2; Illumina). Rank invariant normalization was used to remove or minimize non-biological systematic variation. Differences in gene expression between TINT, tumor, or cell line samples and normal prostate control reference samples were compared using the Mann-Whitney U test. *P*-values for each gene were adjusted to minimize false-positive results by using the Benjamini and Hochberg procedure. To examine similarities in gene expression in the different samples, we performed average linkage clustering with Pearson correlation on the whole dataset of 35 samples (8 tumors, 11 TINT, 15 normal prostate controls, and 1 AT-1 cell line).

Next, fold changes in gene expression were calculated by dividing the mean signal for each probe in the TINT group by the mean signal for each probe in the control group. A list of differentially expressed candidate genes in TINT vs. control prostate tissue was created by selection of those that had (a) a *p*-value of < 0.05, (b) ≥ 2-fold variation in expression, and (c) a probe signal of at least twice the background signal in at least one of the two groups.

Genes that were significantly differently expressed in TINT compared to control prostate, and also their corresponding expression in AT-1 tumor tissue, were visualized in a heatmap using MultiExperiment Viewer software (MeV version 4.9; www.tm4.org). Hierarchical gene clustering was performed using average linkage clustering with Pearson correlation. The genes that were significantly expressed in TINT were further analyzed for enriched biological processes and pathways using GeneGo MetaCore software (www.genego.com). GeneGo software includes a manually annotated database of biological pathways and processes obtained from the scientific literature. The software uses algorithms to create lists of networks and pathways, ranked according to calculated statistical significance (https://portal.genego.com/help/P-value_calculations.pdf).

### Real-time RT-PCR

RT-PCR was used to confirm some of the microarray data. Eleven genes (*Hmox1*, *Lox*, *Cd68*, *Lpl*, *Cebp-beta*, *Cyr61*, *Mmp3*, *S100a4*, *Tgf-bi*, *Mme*, *and Gtsm1*) that represent different clusters (see below) were selected for validation. *Psmc4* was used as a reference gene. Total RNA was extracted as above and treated with DNA-*free* Kit (Ambion, Austin, TX) to remove DNA contamination and reverse transcription was performed using superscript III (Invitrogen, Carlsbad, CA). Taqman assays with gene-specific primers and probes set (Applied Biosystems, Foster City, CA) for each gene were used (*Hmox1*: Rn01536933_m1, *Lox*: Rn01491829_m1, *Cd68*: Rn01495634_g1, *Lpl*: Rn00561482_m1, *Mmp3*: Rn00591740_m1, *Gstm1*: Rn00755117_m1, *Cebp-beta*: Rn00824635_s1, *Cyr61*: Rn01523136_g1, *S100a4*: Rn01451938_m1, *Tgf-bi*: Rn01442102_m1, *Mme*: Rn00561572_m1, and *Psmc4*: Rn00821599_g1). The Mann-Whitney U test was used for comparisons between groups and any *p*-value *<* 0.05 was considered significant.

## Results and Discussion

### TINT-changes are different from those induced by injection of tissue culture medium or heat-killed tumor cells

In order to study the effect of a tumor on the surrounding normal prostate tissue (TINT), we implanted rat AT-1 prostate tumor cells into the prostates of immune-competent rats and sacrificed the animals at day 10 when the tumors were still surrounded by normal prostate tissue. In this animal model, TINT is the tumor-adjacent non-malignant rat prostate tissue, which contains both morphologically normal-appearing epithelium and stroma (S1 Fig).

As we injected presumably antigenic cells into fully immune-competent syngenic animals this should induce an acute immune response. This reaction could be unspecific and its magnitude could be largely unrelated to the presence of a growing tumor, particularly as the time-point when we were able to examine TINT was early after tumor cell injection (already at day 10 the tumors occupied 64 ± 28% (SD), *n* = 11 of the whole prostate lobe). However, the host response to a tumor in experimental models and in patients is strikingly similar to an inflammation-like wound-response [3, 5, 16–19]. Tumors actually exploit their capacity to induce an inflammatory reaction (characterized by accumulation of inflammatory cells, angiogenesis and altered extracellular matrix), by secreting factors that reeducate the accumulating inflammatory cells to support tumor growth [20, 21].

We therefore first considered whether our controls were appropriate before drawing any conclusions on how tumors may influence the tumor-bearing organ. By comparing and quantifying the normal prostate tissue reaction to local injection of RPMI medium, heat-killed tumor cells or to growing tumors, we conclude that the prostate tissue response to medium is very discrete and similar to that of heat-killed tumor cells and that these responses are of considerably lower magnitude that those induced by growing tumors (Table 1). Additionally, the morphology of the control injected prostate lobes was similar to that in the corresponding contralateral non-injected lobes (data not shown). Against this background we consider it appropriate to use RPMI-injected prostates as a control when describing tumor-induced gene expression changes in prostate tissue. Particularly as qRT-PCR examination showed only discrete differences in gene expression between RPMI and heat-killed tumor cell injected prostates, and that gene expression in TINT was different from that in both these controls (see below).

**Table 1:** Quantification of morphological changes in the tumor-bearing organ (TINT-changes) in rats carrying AT-1 tumors and in different controls.

	AT-1 tumors	RPMI-medium controls	Heat-killed AT-1 controls
**Volume density of CD68** ^**+**^ **cells (%)**	1.1 +/- 0.46^a^	0.18 +/- 0.075	0.18+/-0.094^b^
**Volume density of blood vessels (%)**	1.9 +/- 0.34 ^a^	0.86 +/- 0.20	0.75+/-0.26 ^b^
**Endothelial BrdU- labeling index**	23 +/- 5.1 ^a^	1.3 +/- 0.45	1.5+/-0.64 ^b^
**Volume density of Mast cells (%)**	0.34 +/- 0.10 ^a^	0.20 +/- 0.10	0.20 +/-0.060 ^b^

Values are means +/- SD, *n* = 7–9 animals in each group.

^a^ Significantly different than in both controls,

^b^ Not significantly different from that in RPMI injected animals, *p* < 0.05

### The presence of a prostate tumor alters gene expression in the surrounding tumor-bearing organ

**Fig 1:**
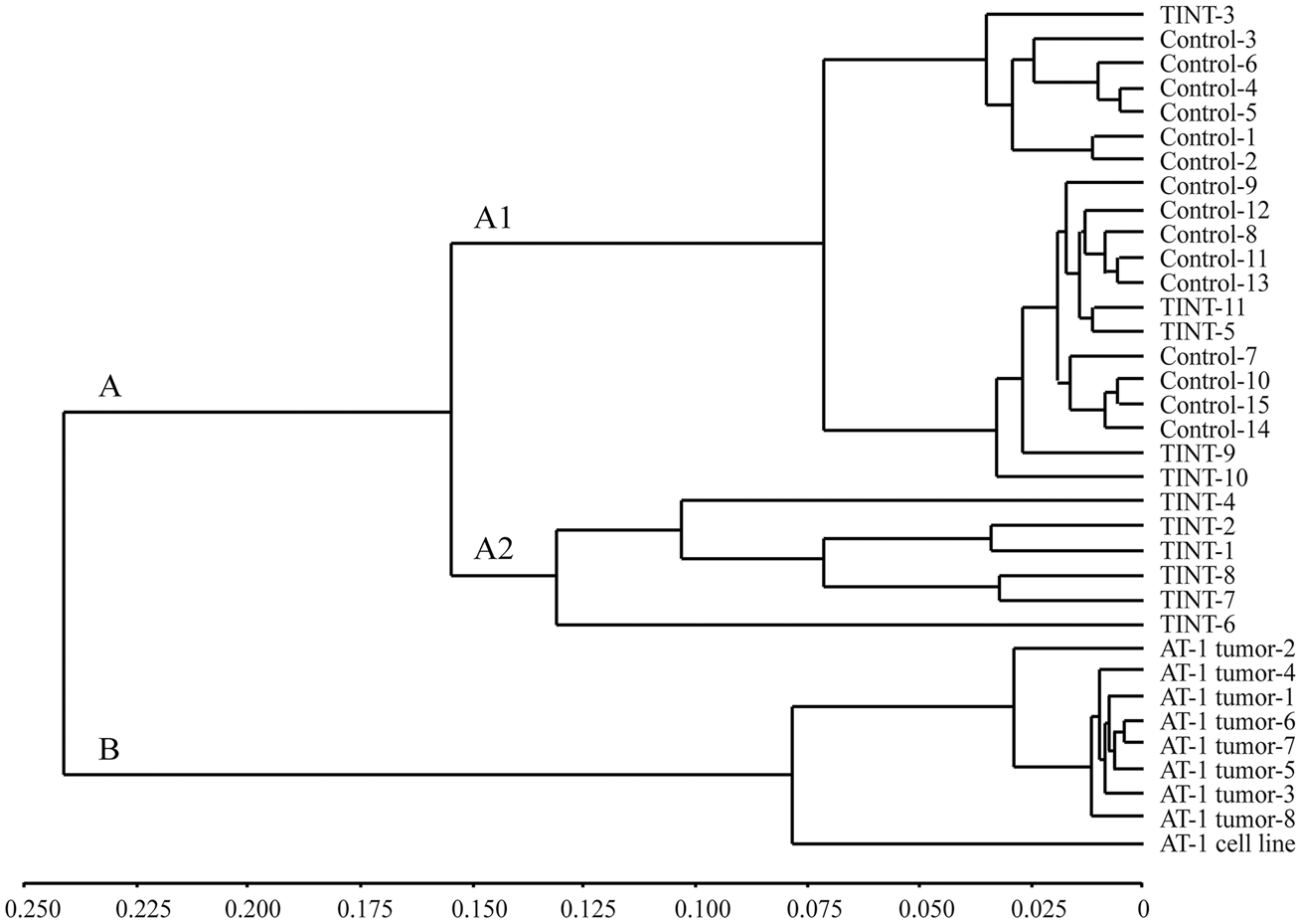
Unsupervised hierarchical clustering of samples based on the genome-wide expression profiles. Control-, TINT-, tumor samples, and the AT-1 cell line were clustered based on the entire microarray dataset. The dendogram shows two major groups; A) containing TINT- and normal prostate tissue samples, and B) containing all AT-1 tumor tissue samples and the AT-1 cell line. TINT: tumor-adjacent normal prostate tissue.

A genome-wide expression microarray was used to compare gene expression in TINT and AT-1 tumor tissue to that in normal control prostate tissue (RPMI injected) from tumor-free animals. Unsupervised hierarchical clustering based on the genome-wide expression profiles clustered the samples in two major groups: (A) all TINT samples and normal prostate tissue samples, and (B) all AT-1 tumor tissue samples and the AT-1 cell line (Fig 1). Gene expression in tumor tissues was therefore clearly separate from the gene expression in normal prostate tissue and TINT. Group A was further subdivided into a group with only TINT tissue (A-2) and a group with control prostate tissue and a few TINT samples (A-1) (Fig 1). This difference in clustering of the TINT samples could not be explained by differences in tumor size. Further studies are required to examine why gene expression in some TINT samples is more affected than in others.

In this study we are not able to completely exclude that single tumor cells could be present in the micro-dissected TINT tissue. However, the AT-1 tumors grew as single rounded tumors with well defined borders and dissection with a margin > 0.5 mm therefore likely avoided tumor cell contamination (S1 Fig). Particularly as we never observed small clusters of AT1 cells, separated from the main tumor, in our immune-stained and extensively examined paraffin embedded tissue samples. As the gene expression profile in AT-1 tumors *in vivo* was determined we could also simulate (in-silico) AT-1 tumor contamination of different degrees in our normal samples. Adding from 0.1 to 20% of the average gene expression signals in tumor tissue to all our individual normal control samples did not make their gene expression pattern TINT-like and the individual samples still clustered at the same position as before (i.e. individual samples initially found in group A1 did not move to group A2, data not shown). This and the finding that expressions of several genes were larger in TINT than in both tumor and normal tissue (see below) suggest that the TINT gene expression pattern is unlikely to be explained by tumor cell contamination. However, it should be noted that we cannot completely ensure that TINT samples were 100% tumor free.

### Genes differentially expressed in TINT compared to normal prostate control tissue suggest that there is an inflammatory and matrix-reorganizing response in the tumor-bearing organ

We identified 5,888 genes with significantly different expression in TINT compared to control samples (*p* < 0.05, data not shown). To identify strong candidate genes that characterize TINT, we selected genes with ≥ 2-fold change, *p* < 0.05, and a probe signal at least twice the background signal in at least one of the two groups.

A total of 461 genes were identified; of these, expression of 423 genes was up-regulated and expression of 38 was down-regulated in TINT relative to normal prostate tissue (S1 Table). In addition, gene expression of the 461 candidate genes in AT-1 tumor tissue was compared to normal prostate control tissue using the same procedure (S1 Table). To characterize TINT and determine what biological processes the 461 candidate genes selected were associated with, we preformed a gene ontology analysis using GeneGo MetaCore software. As could be predicted from our previous findings in this rat model (see Introduction), many of the genes altered in TINT were related to processes such as inflammatory responses and organization of the extracellular matrix (ECM) (Table 2).

**Table 2:** Ontology analysis of 461 TINT candidate genes.

	P-value	Ratio
**Processes**		
Extracellular matrix organization	3.05E-30	(54/407)
Inflammatory response	4.19E-24	(54/593)
Blood coagulation	1.29E-22	(61/665)
Immune response	7.49E-22	(55/1505)
Innate immune response	8.09E-20	(65/983)
**Networks**		
Cell adhesion, Cell-matrix interactions	2.65E-16	(39/211)
Cell adhesion, Platelet-endothelium-leucocyte interactions	1.77E-10	(28/174)
Proteolysis, ECM remodeling	4.17E-09	(18/85)
Cell adhesion, Integrin-mediated cell-matrix adhesion	2.15E-08	(28/214)
Development, Blood vessel morphogenesis	3.15E-07	(27/228)
**Pathways**		
Cell adhesion, ECM remodeling	3.19E-11	(14/52)
Immune response, Antigen presentation by MHC class II	5.21E-09	(7/12)
Immune response, CCR3 signaling in eosinophils	7.43E-08	(13/77)
Cell cycle, Spindle assembly and chromosome separation	1.04E-07	(9/33)
Cell cycle, Role of APC in cell cycle regulation	1.14E-06	(8/32)

Ratio: significantly altered genes in the experiment of all genes in the respective process/network/pathway.

Irrespective of the obvious differences between a short-term rat tumor-implantation model and patients, our studies in prostate cancer patients suggest that some of the changes in TINT seen in the rat model are also present in and related to tumor aggressiveness in patients—for example, increased vascular density, accumulation of inflammatory cells, and alterations in the extracellular matrix [1, 8, 9, 13, 22]. Altered gene expression in histologically normal tumor-adjacent tissue relative to that in normal prostate tissue from men without prostate cancer has also been found in other studies [17, 23–26]. Collectively, they all show similarities in gene expression between tumor-adjacent prostate tissue and tumor tissue. Importantly, and in line with our rat model, tumor-adjacent prostate tissue in patients is characterized by processes such as inflammation and wounding (see below) [17]. Gene expression in tumor-adjacent tissue in breast cancer patients is also characterized as a wounding response [27].

### Changes in TINT could be induced by tumor-secreted factors and tissue hypoxia

Some of the changes in gene expression in TINT are probably due to signals coming from the tumor to the surrounding tumor-bearing organ. Such signals may be soluble factors and/or microvesicles/exosomes [28]. For example, prostate tumor epithelial cells secrete factors that attract inflammatory cells [7, 29–32] and the TINT in turn expressed macrophage chemo-attractants such as chemokine (C-C) ligand 2 (*Ccl2*) and colony-stimulating factor 1(*Csf1*). Expression of genes encoding markers of macrophages (*Cd68*) particularly of the tumor-stimulating “M2 type” (*Cd163*, *Mrc1*, *Mgl1*, *Folr2*, and *Hmox1*), lymphocytes (*Cd8a*), and mast cells (the mast cell chymase gene *Cma1* and the gene for mast cell antigen 32, *Mca32*) was thus upregulated in TINT.

One key factor that induces changes in stroma cells in wounds and tumors is transforming growth factor β1 (*Tgfb1*) [33] and as *Tgfb1* mRNA expression was increased in TINT and tumor tissue (S1 Table), it could be an important inducer of TINT. Other regulatory systems of importance for prostate development and growth such as the Wnt- [34] and Slit-robo systems [35] could also be involved. Genes coupled to these systems and whose expression was found to be upregulated in TINT include *Sfrp2*, *Slit3*, *Robo*-1, and the gene for disabled-2 (*Dab2*). SFRP2 is an androgen-regulated inhibitor of Wnt signalling that is important during prostate development [34]. SLIT3, which is produced by the prostate stroma and stimulated by androgens, is important both during prostate development and for prostate cancer progression [34, 35]. DAB2 is a modulator of signalling downstream of the androgen receptor AR [36]. Interestingly, expression of *Bcl2a1* was also found to be upregulated in TINT, possibly making the tissue more resistant to apoptosis. Altered AR function and up-regulation of expression of anti-apoptotic factors might explain the blunted response to castration in TINT prostate [6].

Prostate tumor epithelial cells also secrete factors that affect the fibromuscular stroma and ECM within the tumor and in the surrounding TINT [1, 37]. In line with this, we found here that expression of genes encoding stroma-related factors, such as *S100A4*, periostin (*Postn_perdicted*), *Sparc*, *CXCL12*, various collagens (*Col1a1*, *Col1a2*, *Col3a1*, *Col4a1*, *Col5a1*, *Col5a2*, *Col6a1_predicted*, *Col6a3_predicted*, *Col8a1_predicted*, *Col14a1_predicted*, *Col15a*), vimentin (*Vim*), elastin (*Eln*), fibronectin (*Fn1*), and lysyl oxidase (*Lox*) was higher in TINT than in control tissue. Similarities between tumor stroma and TINT stroma [37] may suggest common underlying mechanisms. We therefore suggest that tumor cells send signals to both the adjacent tumor stroma and further away to the tumor-bearing organ to facilitate subsequent tumor growth. Some of these signals probably also have systemic effects. Changes seen in TINT have also been described in pre-metastatic niches, for example up-regulation of genes encoding LOX, fibronectin, periostin, MMPs, and S100 proteins [5, 28, 38], suggesting that they could be caused by similar signals from the tumor.

**Fig 2:**
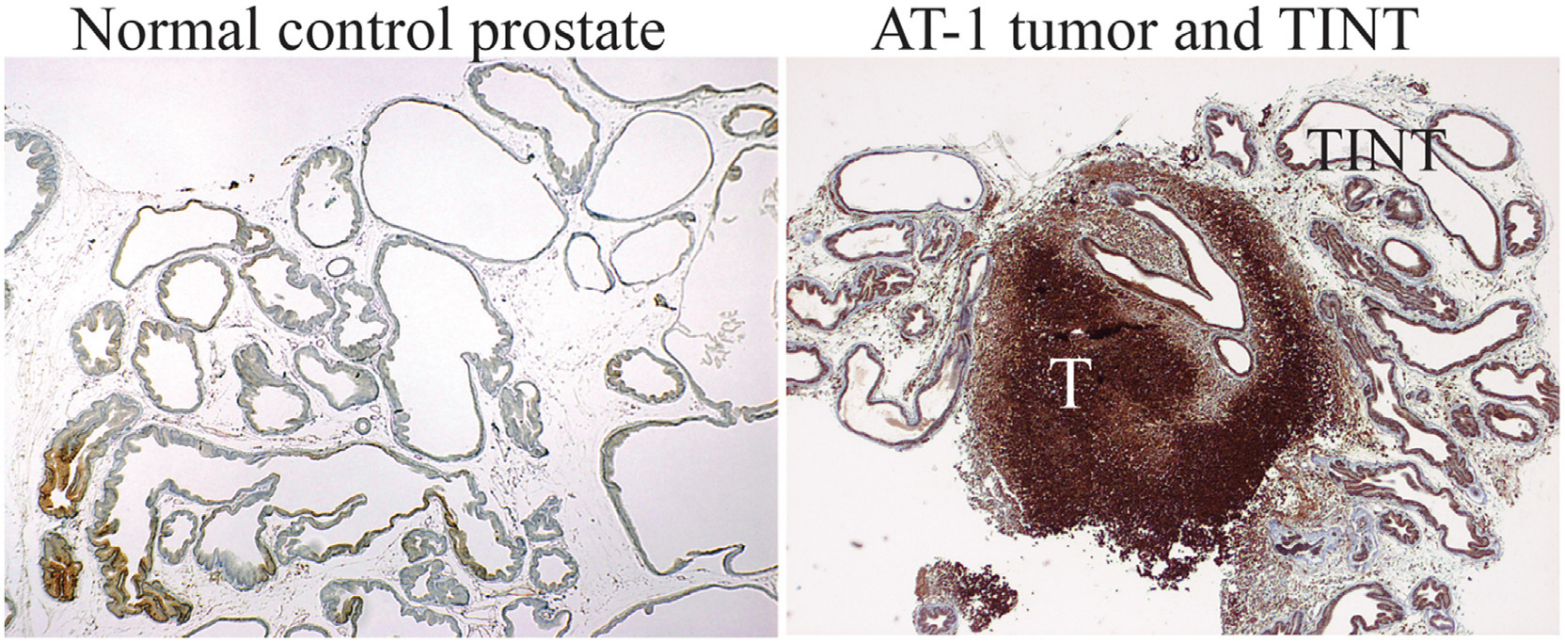
Hypoxic areas in tumor and TINT tissues. Pimidazole staining (brown) of hypoxic tissue areas in normal sham injected control prostate tissue, in AT-1 tumors (T), and in the tumor-adjacent normal prostate tissue (TINT) and at day 10.

Rapid growth of a tumor inside the prostate may also result in some degree of hypoxia in the surrounding normal tissue. As many of the genes whose expression is upregulated in TINT relative to normal prostate tissue are hypoxia-regulated—for example, *Hmox*, *Lox*, osteopontin (*Spp1*), periostin (*Postn_perdicted*), and stroma-derived factor 1 (*Cxcl12/Sdf-1*), we evaluated prostate tissue hypoxia using Hypoxyprobe. This marker identifies cells with a pO_2_ below 10 mmHg. In the AT-1 tumors, large areas of tumor epithelial cells were stained as previously described [6, 7]. The most intense staining was observed in areas remote from visible blood vessels, whereas cells adjacent to vessels were largely unstained (Fig 2). In the tumor-adjacent tissue, some of the normal prostate epithelial cells also showed staining (Fig 2) and the percentage of hypoxic prostate epithelial cells in TINT was larger than in medium injected controls (8.6 ± 2.8% vs. 1.8 ± 1.4%; *n* = 7 to 10 in each group, *p* < 0.001, Mann-Whitney U- test). Although most of the hypoxia regulation of HIF does not occur at the mRNA level [39], *Hif-1α* expression was upregulated 1.7-fold in TINT relative to normal prostate tissue (*p* = 0.01). Hypoxia is therefore a likely underlying mechanism for some of the up-regulation of genes in TINT.

### Gene expression patterns characterizing TINT

**Fig 3:**
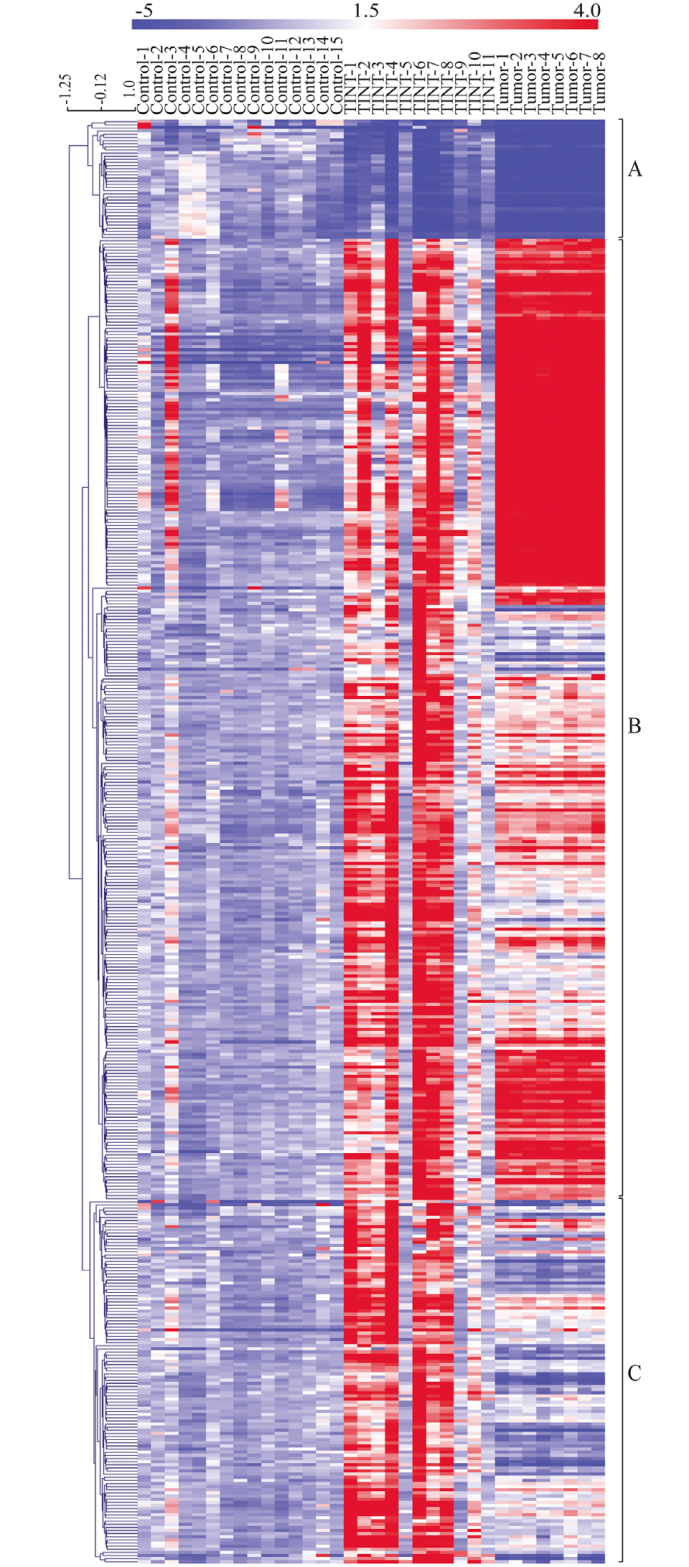
Heatmap showing the relative expression levels of genes differentially expressed between TINT and controls. The rows represent expression levels for each of the 461 genes that were differentially expressed between TINT and normal prostate control tissue and the columns represent the signature of each sample (normal, TINT and tumor). Red color illustrates higher expression than the mean expression in normal prostate tissue and blue illustrates lower expression. Genes were hierarchical clustered using average linkage clustering with a pearson correlation. Three major gene clusters were identified: A) genes down-regulated in TINT and tumour vs. normal tissue, B) genes mainly up-regulated in both TINT and tumor tissue vs. normal tissue and C) genes exclusively up-regulated in TINT vs. normal control tissue. TINT: tumor-adjacent normal prostate tissue.

Furthermore, the differential expression in TINT relative to that in normal prostate tissue, with 461 significantly altered genes, was visualized in a clustering-based heatmap (Fig 3). The heatmap also included the expression levels of selected candidate genes in AT-1 tumor tissue relative to controls. Hierarchical clustering of all samples resulted in 3 major groups of gene expression profiles (Fig 3).

#### Cluster A: Genes down-regulated in TINT relative to normal controls

The first cluster contained 38 genes that were downregulated in TINT relative to controls (Fig 3). Expression of all of these genes was also found to be reduced in the tumor samples (Fig 3). This shows that tumor implantation can suppress expression of genes that are commonly repressed in tumors also in the surrounding normal tissue. In this group we, for example, find genes such as those for glutathione S-transferases (*Gst*) M1, *Gstm2*, *Gstm3*, *Gstm6*, the membrane metallo-endopeptidase gene (*Mme/Cd10*), and the microseminoprotein gene (*Msmb*) (S2 Fig). Ontological analyses show that the genes whose expression is downregulated in TINT are involved in biological networks such as response to hypoxia and oxidative stress, and pathways such as glutathione metabolism (Table 3).

**Table 3:** Ontology analysis of genes in cluster A, B and C.

	Processes	p- value (Ratio)	Networks	p- value (Ratio)	Pathways	p- value (Ratio)
**Cluster A**	Tonic smooth muscle contraction	1.11E-09 (3/14)	Response to hypoxia and oxidative stress	1.68E-02 (2/163)	Glutathione metabolism, Rodent version	2.24E-04 (3/70)
	Cellular hypotonic response	1.11E-08 (3/6)	Cytoskeleton, Actin filaments	1.94E-02 (2/176)	Cell adhesion, Integrin-mediated cell adhesion and migration	3.11E-03 (2/48)
	Aorta smooth muscle tissue morphogenesis	2.22E-08 (3/6)	Cytoskeleton, Intermediate filaments	9.76E-02 (1/81)	Chemotaxis, Inhibitory action of lipoxins on IL-8- and Leukotriene B4-induced neutrophil migration	3.50E-03 (2/51)
**Cluster B**	Immune system process	8.45E-49 (141/2482)	Cell adhesion, Cell-matrix interactions	4.61E-14 (31/211)	Immune response, Antigen presentation by MHC class II	5.97E-10 (7/12)
	Response to stress	3.55E-46 (174/4024)	Cell adhesion, Leucocyte chemotaxis	1.10E-07 (22/205)	Cell cycle, Spindle assembly and chromosome separation	7.09E-09 (9/33)
	Response to wounding	5.00E-45 (105/1415)	Cell adhesion, Integrin-mediated cell-matrix adhesion	2.36E-07 (22/214)	Cell adhesion, ECM remodeling	3.91E-08 (10/52)
**Cluster C**	Response to wounding	4.27E-15 (36/1415)	Proteolysis, ECM remodeling	1.50E-06 (8/85)	Blood coagulation, Blood coagulation	5.36E-08 (6/39)
	Extracellular matrix organization	1.364E-14 (21/43)	Inflammation, Kallikrein-kinin system	1.50E-06 (11/185)	Expression targets of Tissue factor signaling in cancer	5.25E-06 (4/22)
	Extracellular structure organization	1.43E-14 (21/14)	Blood coagulation	3.23E-06 (8/94)	Cell adhesion, ECM remodeling	8.27E-06 (5/52)

Ratio: significantly altered genes in the experiment of all genes in the respective process/network/pathway.

GTS enzymes protect against DNA damage and hypermethylation. Another GST enzyme, P1, is downregulated in human prostate epithelial cells as a consequence of chronic inflammation. In addition, GST P1 is involved in the pathogenesis of prostate cancer and is commonly due to epigenetic changes downregulated in human prostate tumors [40]. It is therefore plausible that inflammation also results in decreased expression of the other GST enzymes in the tumor-adjacent prostate tissue that we identified in this model.

Microseminoprotein is produced almost exclusively by prostate epithelial cells. In the prostate it is known to be a tumor suppressor, and reduced MSMB expression has been shown to be a potentially useful diagnostic and prognostic biomarker of prostate cancer in tissue and serum [41, 42]. Our results suggest that reduced synthesis of microseminoprotein in TINT could contribute to reduced serum levels in patients with prostate cancer.

In line with our microarray results, although not fully verified by qRT-PCR, CD10 expression has also been shown to be downregulated in the normal prostate tissue adjacent to high-grade (Gleason 8–10) tumors [24]. However, Risk and co-workers found that 18 genes were upregulated and 16 were down-regulated in human tumor-adjacent prostate tissue but none, except *CD10*, were similar to those found in our rat model.

#### Cluster B: Genes altered in both TINT and tumor tissue relative to controls

The second cluster contained 307 genes with altered gene expression in both TINT and tumor samples relative to controls (Figs 3 and S2). The cluster mostly contained genes that had a similar up-regulation in both TINT and tumor tissue and up-regulated genes with higher expression in tumor tissue than in TINT tissue (Figs 3 and S2). The gene cluster is characterized by the involvement of biological processes such as the immune system; stress and wounding responses; biological networks such as cell adhesion, chemotaxis; and pathways such as Immune response, Antigen presentation by MHC class II (Table 3). The processes that lead to formation of tumor stroma show similarities to the stroma of healing wounds [3, 18, 19, 43]. The current data suggest that TINT and tumor stroma could in part be shaped by similar factors.

Three genes of potential importance that were upregulated in both tumors and TINT were osteopontin (*Spp1*), *Tgfb1*, and periostin (*Postn*). Osteopontin is produced by prostate cancer epithelial cells [44] and macrophages, and serves as a signal from tumors to the bone marrow to recruit cancer-supporting stroma cells [3, 16]. Increased osteopontin levels in blood are associated with poor prognosis in prostate cancer [45]. The present study suggests that osteopontin could also be produced by the tumor-adjacent normal prostate tissue. Increased expression and secretion of factors from the tumor-adjacent prostate tissue could thus amplify tumor signals to the circulation. Small but aggressive tumor could by creating a large TINT zone therefore have major systemic effects, for example by secretion of LOX and osteopontin also from the surrounding normal tissue (see [6] for discussion). Additional studies are needed to test this hypothesis and clarify the role of osteopontin in TINT.

Periostin, a TGF-beta and hypoxia-inducible protein whose expression has previously been shown to be upregulated in the stroma of a variety of tumors [46], was also found to be upregulated in rat prostate TINT and tumors. Periostin is mainly produced by prostate tumor stroma cells, and high stoma periostin levels are associated with poor outcome [47–49]. As periostin is an important regulator of several aspects of cancer including EMT [46] and metastatic colonization [38], further studies are needed to investigate its role in TINT of the prostate.

Our results may indicate that current strategies to screen for novel cancer markers are insufficient, as—to date—only factors that are altered in tumors relative to surrounding (normal) tissue have been considered to be potentially useful. However, this strategy may actually overlook some really useful markers, those highly altered in both the tumor and the tumor-bearing organ. Our results suggest that both tumors and TINT may actually share common factors (S2 Fig and Fig 3).

#### Cluster C: Genes that are exclusively up-regulated in TINT

The third cluster contained 116 genes that had greater expression in TINT than in controls and tumor tissue (Fig 3), suggesting that there may be markers that can differentiate TINT from both control prostate and tumor tissue. Some genes had upregulated expression in TINT and down-regulated expression in tumors—for example, the mast cell chymase gene (*Cma1*), the tumor necrosis factor receptor superfamily member 11b (osteoprotegerin) gene (*Tnfrsf11b*), and the secreted frizzled related protein 2 gene (*Sfrp2*). This demonstrates that changes in gene expression in TINT are unlikely to be explained by the presence of tumor cells in the tissue (also see above). Factors that are specifically upregulated in TINT could be functionally important, as they indicate that certain processes have to be changed differently in TINT than in tumors. Ontological analysis showed that this group of genes is characterized by processes such as wounding and organization of the extracellular matrix, networks such as inflammation and blood coagulation, and pathways such as tissue factor signaling in cancer (Table 3).

Many genes in this cluster are associated with inflammation, for example those for macrophage markers (*Cd68*, *Cd163*, and *Hmox1*), those for blood and lymph vessels (*Lyve-1*), and those for extracellular matrix (*Lox)* and blood-coagulation proteins (*Pai1*, *F3*, *F5*, *F13a1*, *A2m*, and *Plat*).

Several individual genes that were found to be differentially expressed in TINT are interesting and deserve special study, as they already have known biological functions in tumor stroma and as signals to remote organs. One highly upregulated factor was heme oxygenase 1 (from the *Hmox1* gene), which has been shown to be regulated by cellular stress caused by for example hypoxia and inflammatory cytokines [50]. As HMOX-1 protects against cell damage, it is possible that some TINT-specific changes may be part of the host defense, but as HMOX-1 produces CO—resulting in vasodilatation and angiogenesis—it could also have tumor-stimulating properties [50]. HMOX-1 has also been shown to facilitate tumor progression by suppressing the tumoral immune response [51]. Separate studies investigating the role of HMOX-1in TINT are in progress.

Lysyl oxidase (LOX) is an enzyme that is also stimulated by TGF-β1 and hypoxia and causes cross-linkage between collagen and elastin (which were also upregulated in TINT) [52–55]. In this way, LOX may increase matrix stiffness and facilitate tumor growth [56]. Primary tumors, in response to hypoxia, secrete LOX that facilitates metastatic establishment by increasing the stiffness in pre-metastatic niches [57, 58]. LOX may therefore promote tumor growth by also increasing stiffness in TINT. Further studies examining LOX in more detail are in progress.

Other factors of possible importance that are upregulated in TINT are alpha-1 macroglobulin (encoded by the *A2m* gene) and alpha-1 anti-chymotrypsin (encoded by *Serpina3n*), *Serpina3n* is upregulated in both TINT and tumor. Both of these proteases, which are produced by prostate epithelial cells, form complexes with PSA and are therefore of importance for both PSA physiology in the prostate and for measuring PSA levels in blood [59]. The observation that their expression may be specifically increased in TINT should therefore be examined in greater detail.

### Conformation of microarray data by qRT-PCR

Real-time PCR was performed to validate some of the observed changes in gene expression in TINT. Eleven of the 461 genes were selected because of their known biological function and importance in prostate tissue (*Hmox1*, *Lox*, *Cd68*, *Lpl*, *Cebp-beta*, *Cyr61*, *Mmp3*, *S100a4*, *Tgf-bi*, *Mme*, *and Gtsm1*). In line with the microarray results, expression of *Hmox1*, *Cd68*, *Cebp-beta*, *Cyr61*, *Tgf-bi*, *Lox*, *Mmp3*, and *S100a4* was found to be higher in TINT than in normal prostate tissue (Table 4). Furthermore, *Gstm1* was conformed to be downregulated in TINT relative to controls (Table 4). However, the expression of the *Lpl and Mme* genes in TINT was not significantly different between groups (Table 4). The degree of variance between the RT-PCR and microarray data is unknown, but the variation seen could be due to the use of different sets of animals. Despite this, nine of eleven genes were conformed to be altered in TINT using qRT-PCR. This shows that the differences in gene expression between TINT and normal prostate tissue that were identified by the microarray analysis were reliable. However, more studies are needed to confirm their expression and significance in prostate cancer patients.

**Table 4:** qRT-PCR validation of selected gene.

Genes	Fold-change TINT vs. RPMI control (microarray)	*p*-value	Fold-change TINT vs. RPMI control (qRT-PCR)	*p*-value	Fold-change heat-killed AT-1 vs. RPMI control (qRT-PCR)	*p*-value
*Lox*	5.2	0.001	3.4	0.021	-1.9	0.043
*Hmox1*	14.5	0.001	50.9	0.000	-1.2	0.290
*Cd68*	4.9	0.007	11.3	0.007	-1.1	0.211
*Lpl*	5.3	0.027	-1.7	0.630	-1.2	0.749
*Cebp-beta*	2.3	0.009	7.8	0.001	-2.6	0.008
*Cyr61*	2.2	0.010	5.4	0.002	1.6	0.298
*Mmp3*	2.6	0.017	5.1	0.003	-3.4	0.013
*S100a4*	5.2	0.003	20.2	0.002	-1.2	0.036
*Tgf-beta*	2.4	0.008	31.3	0.003	-1.3	0.037
*Mme*	-2.0	0.009	-1.7	0.211	-1.5	0.012
*Gstm1*	-2.0	0.001	-1.5	0.016	1.0	0.923

Again, we examined if the choice of control was of importance for gene expression changes in TINT. Expressions of the selected genes in RPMI injected prostates were compared with heat-killed tumor cell injected controls (Table 4). Six of the eleven genes examined had significantly lower expression in prostates with heat-killed AT-1 cells compared to RPMI controls (Table 4), although the difference in fold change was low (-1.2 to -3.4). Five of the six significantly down-regulated genes in heat-killed vs. RPMI controls, were actually upregulated in TINT compared to RPMI controls (Table 4). The remaining 5 genes were not significantly different between the two control groups. The morphological changes (Table 1) and the changes in gene expression pattern in TINT thus appear to be related to the presence of a growing tumor and not to the choice of control.

## Conclusion

Using an animal model, we have shown that histologically normal prostate tissue adjacent to a tumor has a unique gene expression signature relative to normal tumor-free prostate tissue. This shows that the presence of a tumor induces changes in gene expression in the surrounding tumor-bearing organ. Studies are now underway to determine the kinetics of this response and how it differs in prostate tissue surrounding tumors with different aggressiveness. The ultimate aim is to identify candidate genes that could possibly serve as novel diagnostic or prognostic markers and/or therapeutic targets for prostate cancer.

## Supporting Information

S1 Fig Orthotopic AT-1 tumor model. 2x10^3^ AT-1 rat prostate tumor cells were injected to the one of the ventral prostate lobes of immune competent Copenhagen rats and tumors were analyzed at day 10. Tumor instructed normal tissue (TINT) is defined as prostate tissue more than 0.5mm from the tumor border.
(TIF)

S2 Fig High resolution Heatmap. (TIF)

S1 Table Candidate genes. List of 461 significantly altered genes in TINT relative to normal prostate tissue.
(DOCX)

## References

[1] Halin S, Hammarsten P, Adamo H, Wikstrom P, Bergh A. Tumor indicating normal tissue could be a new source of diagnostic and prognostic markers for prostate cancer. Expert Opin Med Diagn. 2011;5(1):37–47. Epub 2011/01/01. 10.1517/17530059.2011.540009 . 23484475

[2] Van Neste L, Herman JG, Otto G, Bigley JW, Epstein JI, Van Criekinge W. The epigenetic promise for prostate cancer diagnosis. Prostate. 2012;72(11):1248–61. Epub 2011/12/14. 10.1002/pros.22459 . 22161815

[3] Hanahan D, Weinberg RA. Hallmarks of cancer: the next generation. Cell. 2011;144(5):646–74. Epub 2011/03/08. 10.1016/j.cell.2011.02.013 . 21376230

[4] McAllister SS, Weinberg RA. The tumour-induced systemic environment as a critical regulator of cancer progression and metastasis. Nature cell biology. 2014;16(8):717–27. 10.1038/ncb3015 . 25082194 PMC6220424

[5] Sleeman JP. The metastatic niche and stromal progression. Cancer Metastasis Rev. 2012;31(3–4):429–40. Epub 2012/06/16. 10.1007/s10555-012-9373-9 22699312 PMC3470821

[6] Halin S, Hammarsten P, Wikstrom P, Bergh A. Androgen-insensitive prostate cancer cells transiently respond to castration treatment when growing in an androgen-dependent prostate environment. Prostate. 2007;67(4):370–7. Epub 2006/12/29. 10.1002/pros.20473 . 17192959

[7] Halin S, Rudolfsson SH, Van Rooijen N, Bergh A. Extratumoral macrophages promote tumor and vascular growth in an orthotopic rat prostate tumor model. Neoplasia. 2009;11(2):177–86. Epub 2009/01/30. 19177202 10.1593/neo.81338 PMC2631142

[8] Johansson A, Rudolfsson S, Hammarsten P, Halin S, Pietras K, Jones J, et al Mast cells are novel independent prognostic markers in prostate cancer and represent a target for therapy. Am J Pathol. 2010;177(2):1031–41. Epub 2010/07/10. 10.2353/ajpath.2010.100070 20616342 PMC2913352

[9] Josefsson A, Adamo H, Hammarsten P, Granfors T, Stattin P, Egevad L, et al Prostate cancer increases hyaluronan in surrounding nonmalignant stroma, and this response is associated with tumor growth and an unfavorable outcome. Am J Pathol. 2011;179(4):1961–8. Epub 2011/08/23. 10.1016/j.ajpath.2011.06.005 21854754 PMC3181394

[10] Wikstrom P, Marusic J, Stattin P, Bergh A. Low stroma androgen receptor level in normal and tumor prostate tissue is related to poor outcome in prostate cancer patients. Prostate. 2009;69(8):799–809. Epub 2009/02/04. 10.1002/pros.20927 . 19189305

[11] Hammarsten P, Karalija A, Josefsson A, Rudolfsson SH, Wikstrom P, Egevad L, et al Low levels of phosphorylated epidermal growth factor receptor in nonmalignant and malignant prostate tissue predict favorable outcome in prostate cancer patients. Clin Cancer Res. 2010;16(4):1245–55. Epub 2010/02/11. 10.1158/1078-0432.CCR-09-0103 . 20145160

[12] Hagglof C, Hammarsten P, Josefsson A, Stattin P, Paulsson J, Bergh A, et al Stromal PDGFRbeta expression in prostate tumors and non-malignant prostate tissue predicts prostate cancer survival. PloS one. 2010;5(5):e10747 Epub 2010/05/28. 10.1371/journal.pone.0010747 20505768 PMC2873980

[13] Josefsson A, Wikstrom P, Egevad L, Granfors T, Karlberg L, Stattin P, et al Low endoglin vascular density and Ki67 index in Gleason score 6 tumours may identify prostate cancer patients suitable for surveillance. Scand J Urol Nephrol. 2012;46(4):247–57. Epub 2012/03/29. 10.3109/00365599.2012.669791 . 22452635

[14] Stenman K, Stattin P, Stenlund H, Riklund K, Grobner G, Bergh A. H HRMAS NMR Derived Bio-markers Related to Tumor Grade, Tumor Cell Fraction, and Cell Proliferation in Prostate Tissue Samples. Biomark Insights. 2011;6:39–47. Epub 2011/04/19. 10.4137/BMI.S6794 21499438 PMC3076017

[15] Isaacs JT, Isaacs WB, Feitz WF, Scheres J. Establishment and characterization of seven Dunning rat prostatic cancer cell lines and their use in developing methods for predicting metastatic abilities of prostatic cancers. Prostate. 1986;9(3):261–81. Epub 1986/01/01. . 3774632 10.1002/pros.2990090306

[16] McAllister SS, Gifford AM, Greiner AL, Kelleher SP, Saelzler MP, Ince TA, et al Systemic endocrine instigation of indolent tumor growth requires osteopontin. Cell. 2008;133(6):994–1005. 10.1016/j.cell.2008.04.045 . 18555776 PMC4121664

[17] Chandran UR, Dhir R, Ma C, Michalopoulos G, Becich M, Gilbertson J. Differences in gene expression in prostate cancer, normal appearing prostate tissue adjacent to cancer and prostate tissue from cancer free organ donors. BMC Cancer. 2005;5:45 10.1186/1471-2407-5-45 15892885 PMC1173092

[18] Dvorak HF. Tumors: wounds that do not heal. Similarities between tumor stroma generation and wound healing. The New England journal of medicine. 1986;315(26):1650–9. 10.1056/NEJM198612253152606 . 3537791

[19] Schafer M, Werner S. Cancer as an overhealing wound: an old hypothesis revisited. Nat Rev Mol Cell Biol. 2008;9(8):628–38. 10.1038/nrm2455 . 18628784

[20] Qian BZ, Pollard JW. Macrophage diversity enhances tumor progression and metastasis. Cell. 2010;141(1):39–51. 10.1016/j.cell.2010.03.014 . 20371344 PMC4994190

[21] Quail DF, Joyce JA. Microenvironmental regulation of tumor progression and metastasis. Nature medicine. 2013;19(11):1423–37. 10.1038/nm.3394 24202395 PMC3954707

[22] Tidehag V, Hammarsten P, Egevad L, Granfors T, Stattin P, Leanderson T, et al High density of S100A9 positive inflammatory cells in prostate cancer stroma is associated with poor outcome. Eur J Cancer 2014;in press 10.1016/j.ejca.2014.03.278 24726733

[23] Kosari F, Cheville JC, Ida CM, Karnes RJ, Leontovich AA, Sebo TJ, et al Shared gene expression alterations in prostate cancer and histologically benign prostate from patients with prostate cancer. Am J Pathol. 2012;181(1):34–42. 10.1016/j.ajpath.2012.03.043 22640805 PMC3388167

[24] Risk MC, Knudsen BS, Coleman I, Dumpit RF, Kristal AR, LeMeur N, et al Differential gene expression in benign prostate epithelium of men with and without prostate cancer: evidence for a prostate cancer field effect. Clin Cancer Res. 2010;16(22):5414–23. 10.1158/1078-0432.CCR-10-0272 20935156 PMC2992073

[25] Rizzi F, Belloni L, Crafa P, Lazzaretti M, Remondini D, Ferretti S, et al A novel gene signature for molecular diagnosis of human prostate cancer by RT-qPCR. PloS one. 2008;3(10):e3617 10.1371/journal.pone.0003617 18974881 PMC2570792

[26] Yu YP, Landsittel D, Jing L, Nelson J, Ren B, Liu L, et al Gene expression alterations in prostate cancer predicting tumor aggression and preceding development of malignancy. J Clin Oncol. 2004;22(14):2790–9. 10.1200/JCO.2004.05.158 . 15254046

[27] Troester MA, Lee MH, Carter M, Fan C, Cowan DW, Perez ER, et al Activation of host wound responses in breast cancer microenvironment. Clin Cancer Res. 2009;15(22):7020–8. 10.1158/1078-0432.CCR-09-1126 19887484 PMC2783932

[28] Peinado H, Lavotshkin S, Lyden D. The secreted factors responsible for pre-metastatic niche formation: old sayings and new thoughts. Semin Cancer Biol. 2011;21(2):139–46. 10.1016/j.semcancer.2011.01.002 . 21251983

[29] Vindrieux D, Escobar P, Lazennec G. Emerging roles of chemokines in prostate cancer. Endocr Relat Cancer. 2009;16(3):663–73. 10.1677/ERC-09-0109 . 19556286

[30] Sfanos KS, De Marzo AM. Prostate cancer and inflammation: the evidence. Histopathology. 2012;60(1):199–215. 10.1111/j.1365-2559.2011.04033.x . 22212087 PMC4029103

[31] Macoska JA. Chemokines and BPH/LUTS. Differentiation. 2011;82(4–5):253–60. 10.1016/j.diff.2011.04.003 21600689 PMC3161128

[32] Zhang J, Patel L, Pienta KJ. CC chemokine ligand 2 (CCL2) promotes prostate cancer tumorigenesis and metastasis. Cytokine Growth Factor Rev. 2010;21(1):41–8. Epub 2009/12/17. 10.1016/j.cytogfr.2009.11.009 S1359-6101(09)00116-6 [pii]. 20005149 PMC2857769

[33] Drabsch Y, ten Dijke P. TGF-beta signalling and its role in cancer progression and metastasis. Cancer Metastasis Rev. 2012;31(3–4):553–68. Epub 2012/06/21. 10.1007/s10555-012-9375-7 . 22714591

[34] Pritchard CC, Nelson PS. Gene expression profiling in the developing prostate. Differentiation. 2008;76(6):624–40. 10.1111/j.1432-0436.2008.00274.x . 18462436

[35] Latil A, Chene L, Cochant-Priollet B, Mangin P, Fournier G, Berthon P, et al Quantification of expression of netrins, slits and their receptors in human prostate tumors. Int J Cancer. 2003;103(3):306–15. Epub 2002/12/10. 10.1002/ijc.10821 . 12471613

[36] Zhoul J, Hernandez G, Tu SW, Huang CL, Tseng CP, Hsieh JT. The role of DOC-2/DAB2 in modulating androgen receptor-mediated cell growth via the nongenomic c-Src-mediated pathway in normal prostatic epithelium and cancer. Cancer Res. 2005;65(21):9906–13. 10.1158/0008-5472.CAN-05-1481 . 16267015

[37] Hagglof C, Bergh A. The stroma-a key regulator in prostate function and malignancy. Cancers (Basel). 2012;4(2):531–48. 10.3390/cancers4020531 24213323 PMC3712705

[38] Malanchi I, Santamaria-Martinez A, Susanto E, Peng H, Lehr HA, Delaloye JF, et al Interactions between cancer stem cells and their niche govern metastatic colonization. Nature. 2012;481(7379):85–9. 10.1038/nature10694 . 22158103

[39] Semenza GL. Hypoxia-inducible factors in physiology and medicine. Cell. 2012;148(3):399–408. 10.1016/j.cell.2012.01.021 22304911 PMC3437543

[40] Nelson WG, De Marzo AM, Yegnasubramanian S. Epigenetic alterations in human prostate cancers. Endocrinology. 2009;150(9):3991–4002. 10.1210/en.2009-0573 19520778 PMC2736081

[41] Dahlman A, Rexhepaj E, Brennan DJ, Gallagher WM, Gaber A, Lindgren A, et al Evaluation of the prognostic significance of MSMB and CRISP3 in prostate cancer using automated image analysis. Mod Pathol. 2011;24(5):708–19. 10.1038/modpathol.2010.238 . 21240253

[42] Whitaker HC, Kote-Jarai Z, Ross-Adams H, Warren AY, Burge J, George A, et al The rs10993994 risk allele for prostate cancer results in clinically relevant changes in microseminoprotein-beta expression in tissue and urine. PloS one. 2010;5(10):e13363 10.1371/journal.pone.0013363 20967219 PMC2954177

[43] Arwert EN, Hoste E, Watt FM. Epithelial stem cells, wound healing and cancer. Nat Rev Cancer. 2012;12(3):170–80. 10.1038/nrc3217 . 22362215

[44] Forootan SS, Foster CS, Aachi VR, Adamson J, Smith PH, Lin K, et al Prognostic significance of osteopontin expression in human prostate cancer. Int J Cancer. 2006;118(9):2255–61. 10.1002/ijc.21619 . 16331611

[45] Thoms JW, Dal Pra A, Anborgh PH, Christensen E, Fleshner N, Menard C, et al Plasma osteopontin as a biomarker of prostate cancer aggression: relationship to risk category and treatment response. Br J Cancer. 2012;107(5):840–6. 10.1038/bjc.2012.345 22871886 PMC3425969

[46] Morra L, Moch H. Periostin expression and epithelial-mesenchymal transition in cancer: a review and an update. Virchows Arch. 2011;459(5):465–75. 10.1007/s00428-011-1151-5 21997759 PMC3205268

[47] Tischler V, Fritzsche FR, Wild PJ, Stephan C, Seifert HH, Riener MO, et al Periostin is up-regulated in high grade and high stage prostate cancer. BMC Cancer. 2010;10:273 10.1186/1471-2407-10-273 20534149 PMC2903527

[48] Tsunoda T, Furusato B, Takashima Y, Ravulapalli S, Dobi A, Srivastava S, et al The increased expression of periostin during early stages of prostate cancer and advanced stages of cancer stroma. Prostate. 2009;69(13):1398–403. 10.1002/pros.20988 . 19479898

[49] Sun C, Zhao X, Xu K, Gong J, Liu W, Ding W, et al Periostin: a promising target of therapeutical intervention for prostate cancer. J Transl Med. 2011;9:99 10.1186/1479-5876-9-99 21714934 PMC3146429

[50] Was H, Dulak J, Jozkowicz A. Heme oxygenase-1 in tumor biology and therapy. Curr Drug Targets. 2010;11(12):1551–70. Epub 2010/08/14. BSP/CDT/E-Pub/00150 [pii]. . 20704546 10.2174/1389450111009011551

[51] Arnold J, Magiera L, Kraman M, Fearon D. Tumoral Immune Suppression by Macrophages Expressing Fibroblast Activation Protein-a and Heme Oxygenase-1. Cancer Immunol Res. 2014;2:121–6. 10.1158/2326-6066.CIR-13-0150 24778275 PMC4007628

[52] Erler JT, Giaccia AJ. Lysyl oxidase mediates hypoxic control of metastasis. Cancer Res. 2006;66(21):10238–41. 10.1158/0008-5472.CAN-06-3197 . 17079439

[53] Denko NC, Fontana LA, Hudson KM, Sutphin PD, Raychaudhuri S, Altman R, et al Investigating hypoxic tumor physiology through gene expression patterns. Oncogene. 2003;22(37):5907–14. Epub 2003/08/30. 10.1038/sj.onc.1206703 1206703 [pii]. . 12947397

[54] Ren C, Yang G, Timme TL, Wheeler TM, Thompson TC. Reduced lysyl oxidase messenger RNA levels in experimental and human prostate cancer. Cancer Res. 1998;58(6):1285–90. Epub 1998/03/27. . 9515817

[55] Taylor MA, Amin JD, Kirschmann DA, Schiemann WP. Lysyl oxidase contributes to mechanotransduction-mediated regulation of transforming growth factor-beta signaling in breast cancer cells. Neoplasia. 2011;13(5):406–18. Epub 2011/05/03. 21532881 10.1593/neo.101086 PMC3084617

[56] Levental KR, Yu H, Kass L, Lakins JN, Egeblad M, Erler JT, et al Matrix crosslinking forces tumor progression by enhancing integrin signaling. Cell. 2009;139(5):891–906. 10.1016/j.cell.2009.10.027 19931152 PMC2788004

[57] Erler JT, Bennewith KL, Nicolau M, Dornhofer N, Kong C, Le QT, et al Lysyl oxidase is essential for hypoxia-induced metastasis. Nature. 2006;440(7088):1222–6. 10.1038/nature04695 . 16642001

[58] Erler JT, Bennewith KL, Cox TR, Lang G, Bird D, Koong A, et al Hypoxia-induced lysyl oxidase is a critical mediator of bone marrow cell recruitment to form the premetastatic niche. Cancer Cell. 2009;15(1):35–44. 10.1016/j.ccr.2008.11.012 19111879 PMC3050620

[59] Lilja H, Cockett AT, Abrahamsson PA. Prostate specific antigen predominantly forms a complex with alpha 1-antichymotrypsin in blood. Implications for procedures to measure prostate specific antigen in serum. Cancer. 1992;70(1 Suppl):230–4. . 1376193 10.1002/1097-0142(19920701)70:1+<230::aid-cncr2820701310>3.0.co;2-y

