# Supplementary figures and images for: Characterization of a Gene Expression Signature in Normal Rat Prostate Tissue Induced by the Presence of a Tumor Elsewhere in the Organ
Source: PLoS One. 2015 Jun 15;10(6):e0130076. doi: 10.1371/journal.pone.0130076 (PMC4468243; doi:10.1371/journal.pone.0130076)

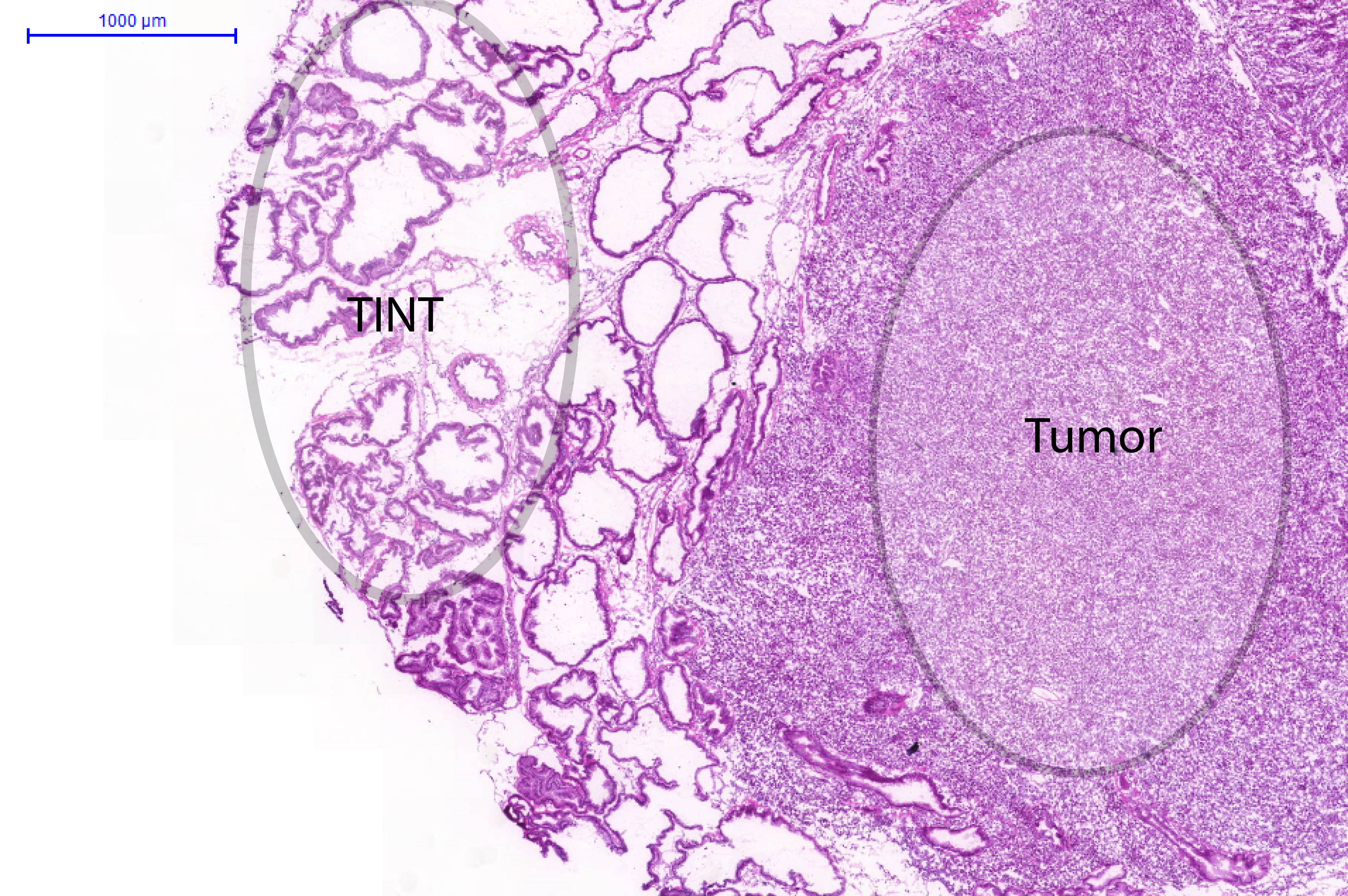

Supplement: S1 Fig — 2x10^3^ AT-1 rat prostate tumor cells were injected to the one of the ventral prostate lobes of immune competent Copenhagen rats and tumors were analyzed at day 10. Tumor instructed normal tissue (TINT) is defined as prostate tissue more than 0.5mm from the tumor border. (TIF) [file pone.0130076.s001.tif]

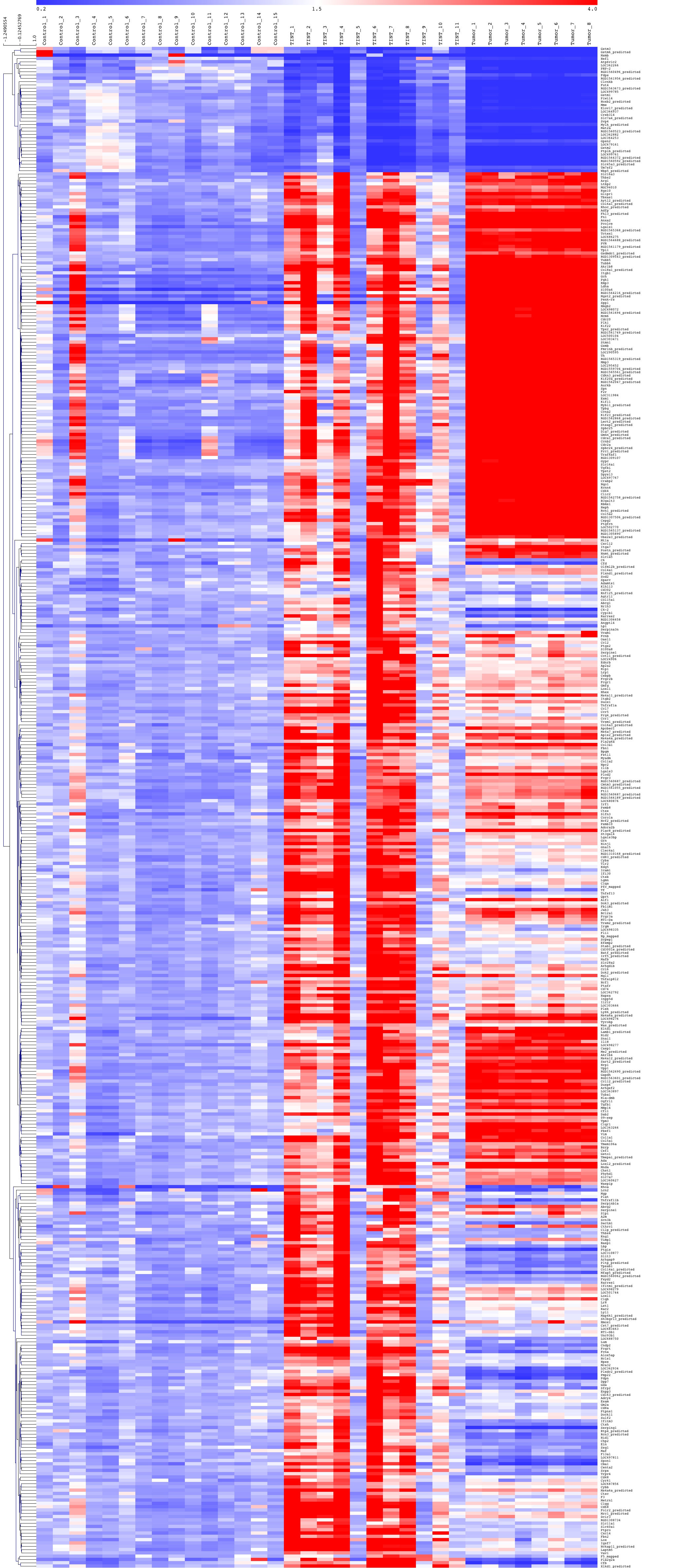

Supplement: S2 Fig — (TIF) [file pone.0130076.s002.tif]
