# Supplementary material for: Characterization of a Gene Expression Signature in Normal Rat Prostate Tissue Induced by the Presence of a Tumor Elsewhere in the Organ
Source: PLoS One. 2015 Jun 15;10(6):e0130076. doi: 10.1371/journal.pone.0130076 (PMC4468243; doi:10.1371/journal.pone.0130076)
Supplement: S1 Table — List of 461 significantly altered genes in TINT relative to normal prostate tissue. (DOCX) [file pone.0130076.s003.docx]

| **Appendix S1** | | | | | |
| --- | --- | --- | --- | --- | --- |
|  | | | | | |
| **Genes downregulated in TINT** | | | | | |
| **Probe_ID** | **Symbol** | **Fold change TINT vs. control** | ***P*-value** | **Fold change tumor vs. control** | ***P*-value** |
| ILMN_1371030 | Atp6v1c2 | -2.0 | 0.01244 | -9.1 | 0.00002 |
| ILMN_1355888 | Clcnkb | -2.0 | 0.02494 | -7.7 | 0.00002 |
| ILMN_1376649 | Creb3l4 | -2.0 | 0.00089 | -20.0 | 0.00002 |
| ILMN_1350899 | Elovl7_predicted | -2.0 | 0.00244 | -4.2 | 0.00002 |
| ILMN_1350983 | Fdps | -2.0 | 0.01373 | -2.6 | 0.00002 |
| ILMN_1361955 | Fut4 | -2.0 | 0.00292 | -25.0 | 0.00002 |
| ILMN_1366848 | Gpsn2 | -2.0 | 0.00919 | -2.3 | 0.00002 |
| ILMN_1365716 | Gstm1 | -2.0 | 0.00147 | -5.6 | 0.00002 |
| ILMN_1350896 | Gstm2 | -2.0 | 0.01136 | -14.3 | 0.00002 |
| ILMN_1358491 | Gstm3 | -2.0 | 0.03348 | -16.7 | 0.00002 |
| ILMN_1356147 | Gstm6_predicted | -2.0 | 0.02748 | -33.3 | 0.00002 |
| ILMN_1360789 | Hoxb2_predicted | -2.0 | 0.00553 | -5.0 | 0.00002 |
| ILMN_1375132 | LOC362266 | -2.0 | 0.01373 | -9.1 | 0.00002 |
| ILMN_1374674 | LOC362882 | -2.5 | 0.00133 | -50.0 | 0.00002 |
| ILMN_1650805 | LOC364253 | -2.0 | 0.01373 | -5.6 | 0.00002 |
| ILMN_1360172 | LOC364937 | -2.0 | 0.00292 | -5.6 | 0.00002 |
| ILMN_1363593 | LOC499761 | -2.0 | 0.01021 | -10.0 | 0.00002 |
| ILMN_1353417 | LOC499785 | -2.0 | 0.00553 | -7.7 | 0.00002 |
| ILMN_1356677 | LOC679161 | -2.0 | 0.00726 | -3.3 | 0.00002 |
| ILMN_1359095 | Mat2a | -2.5 | 0.00292 | -5.0 | 0.00002 |
| ILMN_1352558 | Mme | -2.0 | 0.00919 | -33.3 | 0.00002 |
| ILMN_1349320 | Msmb | -5.0 | 0.02494 | -20.0 | 0.00409 |
| ILMN_1359071 | Mylk_predicted | -2.0 | 0.00147 | -2.9 | 0.00002 |
| ILMN_1361065 | Nefl | -2.0 | 0.01021 | -3.7 | 0.00002 |
| ILMN_1359296 | Piwil4 | -2.0 | 0.00117 | -8.3 | 0.00002 |
| ILMN_1355294 | PRP-2 | -2.0 | 0.03348 | -5.3 | 0.00002 |
| ILMN_1374372 | Ptplb_predicted | -2.0 | 0.01244 | -12.5 | 0.00002 |
| ILMN_1352000 | RGD1560496_predicted | -2.0 | 0.01136 | -10.0 | 0.00002 |
| ILMN_1361968 | RGD1560523_predicted | -2.5 | 0.00244 | -4.8 | 0.00002 |
| ILMN_1368158 | RGD1560592_predicted | -2.0 | 0.03348 | -7.7 | 0.00002 |
| ILMN_1650883 | RGD1561956_predicted | -2.5 | 0.01021 | -4.3 | 0.00002 |
| ILMN_1351389 | RGD1563673_predicted | -2.0 | 0.00823 | -5.3 | 0.00002 |
| ILMN_1348991 | RGD1564372_predicted | -2.0 | 0.01136 | -8.3 | 0.00002 |
| ILMN_1371059 | Slc45a3_predicted | -2.0 | 0.0227 | -5.9 | 0.00002 |
| ILMN_1364763 | Slc7a4_predicted | -2.0 | 0.00244 | -16.7 | 0.00002 |
| ILMN_1368856 | Svp4 | -2.0 | 0.01136 | -20.0 | 0.00002 |
| ILMN_1530410 | Tm7sf2 | -2.0 | 0.00726 | -2.2 | 0.00002 |
| ILMN_1360900 | Wbp5_predicted | -2.0 | 0.01854 | -3.1 | 0.00002 |
| **Genes altered in both TINT and tumor** | | | | | |
| **Probe_ID** | **Symbol** | **Fold change TINT vs. control** | ***P*-value** | **Fold change tumor vs. control** | ***P*-value** |
| ILMN_1354046 | Abcg1 | 3.2 | 0.00483 | 1.4 | 0.00091 |
| ILMN_1358817 | Ada | 2.1 | 0.00205 | 2.3 | 0.00002 |
| ILMN_1367103 | Adamts1 | 2.2 | 0.03348 | 1.0 | 0.83979 |
| ILMN_1357171 | Adfp | 2.3 | 0.03049 | 3.8 | 0.00002 |
| ILMN_1357083 | Adora2b | 2.0 | 0.00345 | 2.1 | 0.00002 |
| ILMN_1356199 | Agtrl1 | 2.5 | 0.03049 | 1.4 | 0.00063 |
| ILMN_1359219 | Aif1 | 5.5 | 0.00175 | 3.9 | 0.00002 |
| ILMN_1363873 | Akr1b4 | 3.8 | 0.00483 | 5.4 | 0.00002 |
| ILMN_1354910 | Akr1b8 | 4.2 | 0.00133 | 9.2 | 0.00002 |
| ILMN_1360466 | Angptl4 | 3.1 | 0.00483 | 1.1 | 0.19057 |
| ILMN_1364260 | Anxa2 | 3.5 | 0.01516 | 6.6 | 0.00002 |
| ILMN_1372527 | Ap1s2_predicted | 4.1 | 0.0008 | 3.6 | 0.00002 |
| ILMN_1353636 | Ap2a2 | 2.3 | 0.00205 | 1.8 | 0.00002 |
| ILMN_1368847 | Apobec1 | 3.2 | 0.00726 | 3.3 | 0.00002 |
| ILMN_1356828 | Arg1 | 2.1 | 0.04051 | 4.2 | 0.00002 |
| ILMN_1372349 | Arhgdib | 3.9 | 0.00147 | 2.1 | 0.00004 |
| ILMN_1376651 | Arhgef2 | 2.8 | 0.00553 | 5.1 | 0.00002 |
| ILMN_1358998 | Aurkb | 2.3 | 0.00726 | 10.6 | 0.00002 |
| ILMN_1357096 | Aytl2_predicted | 3.0 | 0.0041 | 4.2 | 0.00002 |
| ILMN_1356775 | B3galt3 | 2.1 | 0.01854 | 4.6 | 0.00002 |
| ILMN_1368018 | Batf_predicted | 2.1 | 0.01021 | 1.2 | 0.00234 |
| ILMN_1354419 | Bcl2a1 | 4.2 | 0.00553 | 3.6 | 0.00007 |
| ILMN_1362247 | Bpgm | 2.1 | 0.0204 | 1.9 | 0.00002 |
| ILMN_1349787 | Bzrp | 2.3 | 0.01244 | 2.8 | 0.00002 |
| ILMN_1352382 | C1qa | 5.0 | 0.00244 | 2.0 | 0.00029 |
| ILMN_1348939 | C1qr1 | 2.7 | 0.01373 | 6.0 | 0.00002 |
| ILMN_1361837 | C4-2 | 4.2 | 0.00553 | -5.0 | 0.00002 |
| ILMN_1369281 | C6 | 3.6 | 0.01516 | -1.25 | 0.00044 |
| ILMN_1361826 | Casp1 | 2.5 | 0.00147 | 3.2 | 0.00002 |
| ILMN_1368224 | Ccl12_predicted | 2.5 | 0.0041 | 3.6 | 0.00002 |
| ILMN_1357229 | Ccl2 | 4.5 | 0.00483 | 2.4 | 0.00019 |
| ILMN_1369168 | Ccl6 | 5.9 | 0.0008 | 2.0 | 0.00007 |
| ILMN_1366023 | Ccl7 | 9.0 | 0.00133 | 2.1 | 0.00029 |
| ILMN_1353596 | Ccna2 | 2.3 | 0.01021 | 9.0 | 0.00002 |
| ILMN_1360587 | Ccnb2 | 2.9 | 0.01244 | 9.5 | 0.00002 |
| ILMN_1356721 | Ccr1 | 4.0 | 0.00089 | 2.3 | 0.00002 |
| ILMN_1362741 | Ccr5 | 6.0 | 0.00292 | 1.9 | 0.00044 |
| ILMN_1363786 | Cd300le_predicted | 3.9 | 0.00553 | 1.6 | 0.00063 |
| ILMN_1349873 | Cd302 | 2.2 | 0.03049 | 1.1 | 0.83979 |
| ILMN_1352966 | Cd44 | 2.2 | 0.01373 | 6.2 | 0.00002 |
| ILMN_1373953 | Cd74 | 3.1 | 0.01021 | 2.1 | 0.00011 |
| ILMN_1358082 | Cd83_predicted | 2.9 | 0.03348 | 1.9 | 0.00007 |
| ILMN_1373595 | Cdc20 | 2.2 | 0.02748 | 6.7 | 0.00002 |
| ILMN_1358490 | Cdc2a | 2.9 | 0.00919 | 10.7 | 0.00002 |
| ILMN_1375974 | Cdca1_predicted | 2.5 | 0.01136 | 8.2 | 0.00002 |
| ILMN_1364508 | Cdkn3_predicted | 2.7 | 0.00634 | 11.9 | 0.00002 |
| ILMN_1371344 | Cebpb | 2.3 | 0.00919 | 2.1 | 0.00002 |
| ILMN_1371456 | Cfd | 4.9 | 0.01854 | -3.3 | 0.00004 |
| ILMN_1362054 | Cfl1 | 2.0 | 0.00345 | 2.4 | 0.00002 |
| ILMN_1376641 | Chst1 | 2.2 | 0.00634 | 2.2 | 0.00002 |
| ILMN_1376716 | Clec4a1 | 3.4 | 0.00726 | 1.8 | 0.00011 |
| ILMN_1368780 | Clic2 | 2.8 | 0.04885 | 9.6 | 0.00002 |
| ILMN_1649884 | Cmtm3_predicted | 2.6 | 0.00634 | 2.5 | 0.00002 |
| ILMN_1372740 | Col15a1 | 2.5 | 0.04885 | 1.3 | 0.01659 |
| ILMN_1361396 | Col1a1 | 4.6 | 0.00147 | 5.0 | 0.00002 |
| ILMN_1354712 | Col1a2 | 2.4 | 0.00726 | 1.7 | 0.00002 |
| ILMN_1354941 | Col3a1 | 5.2 | 0.00244 | 4.9 | 0.00002 |
| ILMN_1360318 | Col4a1 | 3.8 | 0.01516 | 2.4 | 0.00002 |
| ILMN_1353887 | Col5a1 | 3.1 | 0.01516 | 4.6 | 0.00002 |
| ILMN_1360855 | Col5a2 | 3.8 | 0.00205 | 10.9 | 0.00002 |
| ILMN_1356949 | Col6a1_predicted | 2.9 | 0.01136 | 4.6 | 0.00002 |
| ILMN_1355773 | Col6a3_predicted | 2.3 | 0.03049 | 2.3 | 0.00007 |
| ILMN_1362033 | Col8a1_predicted | 5.9 | 0.00483 | 20.3 | 0.00002 |
| ILMN_1364468 | Coro1a | 3.6 | 0.0041 | 3.2 | 0.00002 |
| ILMN_1358946 | Cotl1_predicted | 5.4 | 0.00133 | 2.7 | 0.00004 |
| ILMN_1367834 | Crabp2 | 3.8 | 0.00244 | 15.2 | 0.00002 |
| ILMN_1363245 | Csf1 | 2.5 | 0.01244 | 3.3 | 0.00002 |
| ILMN_1373952 | Cspg2 | 3.8 | 0.00133 | 10.9 | 0.00002 |
| ILMN_1376513 | Ctsb | 3.8 | 0.00292 | 1.4 | 0.00862 |
| ILMN_1352236 | Ctss | 2.4 | 0.00919 | 2.3 | 0.00002 |
| ILMN_1351603 | Cxcl12 | 2.5 | 0.01373 | 2.5 | 0.00004 |
| ILMN_1366276 | Cyba | 3.7 | 0.00483 | 1.8 | 0.00174 |
| ILMN_1376635 | Cyp1b1 | 2.1 | 0.0167 | -1.25 | 0.00091 |
| ILMN_1355383 | Dab2 | 5.4 | 0.00089 | 7.2 | 0.00002 |
| ILMN_1353258 | Dlg7_predicted | 2.4 | 0.00919 | 7.5 | 0.00002 |
| ILMN_1369416 | Dok2_predicted | 2.8 | 0.00483 | 1.6 | 0.00063 |
| ILMN_1370914 | Dok3_predicted | 2.2 | 0.0204 | 1.7 | 0.00002 |
| ILMN_1374650 | Dpysl3 | 2.2 | 0.00823 | 6.7 | 0.00002 |
| ILMN_1367874 | Duox1 | 3.4 | 0.00069 | 2.6 | 0.00002 |
| ILMN_1362834 | Dusp6 | 2.7 | 0.00726 | 4.7 | 0.00002 |
| ILMN_1372410 | Edg5 | 2.2 | 0.00244 | 1.3 | 0.00063 |
| ILMN_1360909 | Ednrb | 2.5 | 0.00244 | 1.9 | 0.00002 |
| ILMN_1363209 | Efemp2 | 2.8 | 0.00089 | 1.7 | 0.00004 |
| ILMN_1369759 | Eltd1 | 2.1 | 0.04885 | 4.1 | 0.00002 |
| ILMN_1366346 | Emp3 | 3.6 | 0.00634 | 9.5 | 0.00002 |
| ILMN_1360134 | Esm1 | 2.6 | 0.0204 | 17.3 | 0.00002 |
| ILMN_1372371 | F2r | 2.9 | 0.0227 | 10.5 | 0.00002 |
| ILMN_1372951 | Fblim1 | 2.6 | 0.01854 | 2.0 | 0.00011 |
| ILMN_1359541 | Fbn1 | 2.5 | 0.01021 | 2.8 | 0.00002 |
| ILMN_1362025 | Fcgr1 | 2.6 | 0.00205 | 2.2 | 0.00002 |
| ILMN_1353489 | Fcgr2b | 2.1 | 0.00244 | 2.1 | 0.00002 |
| ILMN_1367877 | Fcgr3 | 2.4 | 0.01854 | 2.4 | 0.00044 |
| ILMN_1359086 | Fcgr3a | 3.3 | 0.00205 | 3.0 | 0.00002 |
| ILMN_1351900 | Fcnb | 3.7 | 0.00634 | 4.3 | 0.00002 |
| ILMN_1368369 | Fhl3_predicted | 3.0 | 0.00823 | 5.7 | 0.00002 |
| ILMN_1651013 | Fli1 | 2.1 | 0.0204 | 1.6 | 0.00007 |
| ILMN_1359754 | Fmr1nb_predicted | 2.3 | 0.04885 | 13.7 | 0.00002 |
| ILMN_1348956 | Fn1 | 4.5 | 0.00345 | 8.5 | 0.00002 |
| ILMN_1354884 | Fstl1 | 3.3 | 0.00823 | 2.6 | 0.00011 |
| ILMN_1366981 | Ftl1 | 3.3 | 0.01021 | 3.0 | 0.00007 |
| ILMN_1649859 | Gapdh | 2.3 | 0.0041 | 4.5 | 0.00002 |
| ILMN_1372022 | Gch | 2.4 | 0.00483 | 9.1 | 0.00002 |
| ILMN_1376740 | Glipr1 | 3.3 | 0.01373 | 4.7 | 0.00002 |
| ILMN_1362702 | Gmfg | 3.8 | 0.0008 | 2.5 | 0.00002 |
| ILMN_1374166 | Gmnn_predicted | 2.9 | 0.00483 | 12.2 | 0.00002 |
| ILMN_1358428 | Gna15 | 2.0 | 0.00919 | 1.4 | 0.00002 |
| ILMN_1357411 | Grn | 2.4 | 0.01516 | 1.7 | 0.00004 |
| ILMN_1364932 | Gsdmdc1_predicted | 2.0 | 0.01854 | 3.1 | 0.00002 |
| ILMN_1349038 | Gsto1 | 2.3 | 0.01516 | 3.0 | 0.00002 |
| ILMN_1650650 | Gypc | 2.0 | 0.01373 | 4.2 | 0.00002 |
| ILMN_1358992 | Gzmb | 2.4 | 0.0227 | 20.8 | 0.00002 |
| ILMN_1371544 | Heph | 2.1 | 0.00244 | 5.1 | 0.00002 |
| ILMN_1353851 | Hhex | 2.0 | 0.0167 | 1.7 | 0.00002 |
| ILMN_1375389 | Hip1 | 2.2 | 0.01244 | 1.8 | 0.00011 |
| ILMN_1373278 | Hla-dmb | 3.3 | 0.00175 | 5.1 | 0.00002 |
| ILMN_1372675 | Hmgb2 | 2.2 | 0.01244 | 4.8 | 0.00002 |
| ILMN_1354506 | Icam1 | 2.3 | 0.0227 | 1.0 | 0.91319 |
| ILMN_1357094 | Ifi30 | 3.6 | 0.01136 | 1.3 | 0.01659 |
| ILMN_1352725 | Il18 | 2.4 | 0.00244 | 3.3 | 0.00002 |
| ILMN_1358370 | Il1b | 2.9 | 0.01244 | 2.8 | 0.00002 |
| ILMN_1358643 | Il21r | 2.1 | 0.01516 | 1.5 | 0.00002 |
| ILMN_1350985 | Inpp5d | 2.8 | 0.00175 | 2.0 | 0.00002 |
| ILMN_1372095 | Irf1 | 2.1 | 0.01854 | 2.4 | 0.00002 |
| ILMN_1362902 | Irf5_predicted | 2.7 | 0.0041 | 1.5 | 0.00007 |
| ILMN_1376472 | Irgm | 2.2 | 0.00292 | 1.3 | 0.07843 |
| ILMN_1350665 | Itga7 | 3.1 | 0.04467 | 4.5 | 0.00002 |
| ILMN_1361299 | Itgb1 | 2.3 | 0.04885 | 5.8 | 0.00002 |
| ILMN_1366766 | Itgb2 | 2.5 | 0.00205 | 1.8 | 0.00011 |
| ILMN_1355371 | Jak3 | 4.1 | 0.00634 | 3.6 | 0.00002 |
| ILMN_1364008 | Kcnn4 | 2.6 | 0.01854 | 9.8 | 0.00002 |
| ILMN_1361476 | Kif11 | 2.6 | 0.00553 | 12.6 | 0.00002 |
| ILMN_1370269 | Kif20a_predicted | 2.2 | 0.01244 | 8.8 | 0.00002 |
| ILMN_1358183 | Kif22 | 2.9 | 0.01136 | 8.5 | 0.00002 |
| ILMN_1357437 | Kif23_predicted | 2.4 | 0.00634 | 11.4 | 0.00002 |
| ILMN_1375046 | Klhl13 | 2.5 | 0.00483 | 1.3 | 0.02462 |
| ILMN_1352808 | Lamb1_predicted | 2.5 | 0.03049 | 5.1 | 0.00002 |
| ILMN_1364112 | Ldha | 2.4 | 0.02494 | 5.9 | 0.00011 |
| ILMN_1353461 | Lect2_predicted | 2.3 | 0.01516 | 11.3 | 0.00002 |
| ILMN_1367523 | Lgals1 | 3.8 | 0.03348 | 7.0 | 0.00002 |
| ILMN_1370840 | Lgals3 | 2.1 | 0.0167 | 2.2 | 0.00004 |
| ILMN_1352246 | Lgals3bp | 2.8 | 0.00483 | 1.9 | 0.00007 |
| ILMN_1365385 | Lgmn | 4.9 | 0.00244 | 1.8 | 0.00044 |
| ILMN_1368490 | LOC24906 | 2.4 | 0.00117 | 1.6 | 0.00002 |
| ILMN_1369565 | LOC290595 | 3.1 | 0.00919 | 23.6 | 0.00002 |
| ILMN_1359632 | LOC295452 | 2.0 | 0.01854 | 7.9 | 0.00002 |
| ILMN_1367207 | LOC303471 | 2.7 | 0.00553 | 11.5 | 0.00002 |
| ILMN_1352621 | LOC303666 | 5.0 | 0.00133 | 3.0 | 0.00007 |
| ILMN_1370088 | LOC311984 | 2.0 | 0.00175 | 6.9 | 0.00002 |
| ILMN_1349017 | LOC360627 | 2.8 | 0.00244 | 2.9 | 0.00002 |
| ILMN_1650502 | LOC362792 | 2.0 | 0.01373 | 1.6 | 0.00004 |
| ILMN_1351969 | LOC363266 | 2.3 | 0.00133 | 4.2 | 0.00002 |
| ILMN_1358168 | LOC363897 | 2.0 | 0.00205 | 3.3 | 0.00002 |
| ILMN_1355030 | LOC497767 | 2.2 | 0.00823 | 6.8 | 0.00002 |
| ILMN_1351907 | LOC498072 | 2.1 | 0.01854 | 4.6 | 0.00002 |
| ILMN_1355544 | LOC498276 | 6.2 | 0.00205 | 5.0 | 0.00002 |
| ILMN_1370638 | LOC498277 | 4.7 | 0.00175 | 6.5 | 0.00002 |
| ILMN_1356758 | LOC498335 | 2.8 | 0.00634 | 1.1 | 0.72586 |
| ILMN_1356886 | LOC500104 | 2.1 | 0.0204 | 6.9 | 0.00002 |
| ILMN_1349457 | LOC502770 | 2.5 | 0.00205 | 6.5 | 0.00002 |
| ILMN_1650907 | LOC680876 | 2.2 | 0.0041 | 1.9 | 0.00063 |
| ILMN_1364154 | LOC686275 | 2.5 | 0.0167 | 4.7 | 0.00002 |
| ILMN_1530405 | Loxl1 | 2.7 | 0.00069 | 2.0 | 0.00002 |
| ILMN_1351352 | Loxl2_predicted | 3.9 | 0.00069 | 5.1 | 0.00002 |
| ILMN_1357053 | Lpl | 5.3 | 0.02748 | -2.0 | 0.0108 |
| ILMN_1357522 | Lrp1 | 2.8 | 0.00133 | 2.0 | 0.00002 |
| ILMN_1361640 | Ltbp2 | 2.2 | 0.00292 | 3.5 | 0.00002 |
| ILMN_1358738 | Ly86_predicted | 2.5 | 0.00133 | 2.3 | 0.00002 |
| ILMN_1361027 | Mafb | 3.1 | 0.00345 | 1.4 | 0.00313 |
| ILMN_1356031 | Mcm6 | 2.3 | 0.01244 | 4.5 | 0.00002 |
| ILMN_1351957 | Me2_predicted | 2.4 | 0.00345 | 3.7 | 0.00002 |
| ILMN_1363621 | MGC94010 | 2.2 | 0.01373 | 2.8 | 0.00002 |
| ILMN_1374266 | Mgl1 | 10.2 | 0.0008 | 3.5 | 0.00004 |
| ILMN_1370709 | Mgst2_predicted | 3.9 | 0.02748 | 15.8 | 0.00002 |
| ILMN_1365885 | Mmp14 | 2.7 | 0.01516 | 4.5 | 0.00002 |
| ILMN_1361666 | Mmp3 | 2.6 | 0.0167 | 21.3 | 0.00002 |
| ILMN_2038896 | Mnda | 4.3 | 0.00117 | 5.5 | 0.00002 |
| ILMN_1356010 | Ms4a11_predicted | 6.0 | 0.00147 | 4.4 | 0.00002 |
| ILMN_1367036 | Ms4a12_predicted | 2.3 | 0.00175 | 3.0 | 0.00002 |
| ILMN_1358694 | Ms4a4a_predicted | 4.4 | 0.00205 | 3.5 | 0.00002 |
| ILMN_1373835 | Ms4a6a_predicted | 4.9 | 0.00133 | 3.5 | 0.00002 |
| ILMN_1357510 | Ms4a7_predicted | 2.4 | 0.00244 | 2.0 | 0.00002 |
| ILMN_1367740 | Mt1a | 2.5 | 0.0227 | 2.0 | 0.0068 |
| ILMN_1358691 | Myadm | 2.3 | 0.02748 | 1.8 | 0.00091 |
| ILMN_1363017 | Mybl1_predicted | 2.3 | 0.02748 | 16.3 | 0.00002 |
| ILMN_1374896 | Napsa | 3.9 | 0.00345 | 2.1 | 0.00029 |
| ILMN_1355786 | Ncf1 | 2.9 | 0.00634 | 1.7 | 0.00174 |
| ILMN_1365484 | Ncf2_predicted | 2.3 | 0.00205 | 1.9 | 0.00002 |
| ILMN_1361855 | Nid2 | 2.3 | 0.01136 | 3.7 | 0.00002 |
| ILMN_1354493 | Ninj1 | 3.4 | 0.00634 | 2.1 | 0.00002 |
| ILMN_1351105 | Nnmt_predicted | 3.2 | 0.00483 | 3.8 | 0.00002 |
| ILMN_1368463 | Np_mapped | 2.1 | 0.00147 | 1.4 | 0.00004 |
| ILMN_1352122 | Npc2 | 2.1 | 0.01373 | 1.7 | 0.00019 |
| ILMN_2039396 | Nqo1 | 3.3 | 0.00634 | 10.1 | 0.00002 |
| ILMN_1352599 | Nr1h3 | 2.0 | 0.01373 | 1.2 | 0.00409 |
| ILMN_1367985 | Nrp1 | 3.6 | 0.00089 | 5.2 | 0.00002 |
| ILMN_1376666 | Oasl1 | 2.0 | 0.00919 | 1.9 | 0.00002 |
| ILMN_1368121 | Ogfrl1 | 2.1 | 0.0167 | 2.8 | 0.00002 |
| ILMN_1355412 | Olfml2b_predicted | 2.8 | 0.02494 | 1.9 | 0.00019 |
| ILMN_1363917 | Pbef1 | 2.1 | 0.0167 | 4.3 | 0.00002 |
| ILMN_1373978 | Pcolce | 3.5 | 0.00205 | 7.3 | 0.00002 |
| ILMN_1352410 | Penk-rs | 2.1 | 0.02494 | 8.6 | 0.00002 |
| ILMN_1350897 | Pfc_mapped | 5.3 | 0.00244 | 1.4 | 0.00862 |
| ILMN_1369074 | Pgk1 | 2.4 | 0.01244 | 7.6 | 0.00004 |
| ILMN_1367049 | Phyhd1 | 3.4 | 0.00133 | 2.7 | 0.00002 |
| ILMN_1360060 | Pla2g4a | 2.0 | 0.00069 | 1.9 | 0.00002 |
| ILMN_1363160 | Plac8_predicted | 6.1 | 0.00089 | 6.2 | 0.00002 |
| ILMN_1352911 | Plek | 2.6 | 0.01373 | 2.0 | 0.00007 |
| ILMN_1369432 | Plk1 | 2.0 | 0.01373 | 4.7 | 0.00002 |
| ILMN_1650259 | Plod2 | 3.8 | 0.00205 | 4.7 | 0.00002 |
| ILMN_1365553 | Plxnd1_predicted | 2.4 | 0.03681 | 2.4 | 0.00002 |
| ILMN_1360313 | Postn_predicted | 3.1 | 0.01021 | 4.2 | 0.00002 |
| ILMN_1359126 | Prc1_predicted | 2.8 | 0.00823 | 13.9 | 0.00002 |
| ILMN_1360637 | Prg4_predicted | 2.9 | 0.0008 | 1.3 | 0.00029 |
| ILMN_1358888 | Psmb10 | 2.4 | 0.00147 | 2.2 | 0.00002 |
| ILMN_1362728 | Psmb8 | 2.7 | 0.00919 | 2.9 | 0.00002 |
| ILMN_1354586 | Ptafr | 2.5 | 0.00244 | 2.0 | 0.00002 |
| ILMN_1353023 | Ptgfrn | 2.0 | 0.00726 | 4.7 | 0.00002 |
| ILMN_1349422 | Ptgs2 | 3.0 | 0.00244 | 1.6 | 0.01659 |
| ILMN_1371772 | PVR | 2.1 | 0.0204 | 4.5 | 0.00002 |
| ILMN_1530394 | Qprt | 2.0 | 0.03049 | 1.6 | 0.00007 |
| ILMN_1370339 | Rarres2 | 4.4 | 0.00919 | -2.5 | 0.00002 |
| ILMN_1376410 | Rbms1 | 2.4 | 0.01136 | 5.6 | 0.00002 |
| ILMN_1354661 | Rcn1_predicted | 3.0 | 0.00244 | 8.3 | 0.00002 |
| ILMN_1350828 | RGD1305890 | 2.3 | 0.00553 | 5.1 | 0.00002 |
| ILMN_1650056 | RGD1306658 | 3.5 | 0.00823 | 1.8 | 0.00007 |
| ILMN_1357304 | RGD1307506_predicted | 2.7 | 0.01136 | 6.9 | 0.00002 |
| ILMN_1353170 | RGD1309107 | 3.3 | 0.00483 | 9.9 | 0.00002 |
| ILMN_1361803 | RGD1309543_predicted | 2.2 | 0.03348 | 4.0 | 0.00002 |
| ILMN_1351911 | RGD1310168_predicted | 2.5 | 0.0041 | 1.6 | 0.00007 |
| ILMN_1356078 | RGD1559704_predicted | 2.1 | 0.00823 | 8.6 | 0.00002 |
| ILMN_1367581 | RGD1560687_predicted | 3.9 | 0.01021 | 3.1 | 0.00007 |
| ILMN_1366337 | RGD1560687_predicted | 2.9 | 0.01373 | 2.7 | 0.00002 |
| ILMN_1365578 | RGD1561055_predicted | 3.1 | 0.00175 | 2.7 | 0.00002 |
| ILMN_1365744 | RGD1561179_predicted | 2.5 | 0.03348 | 4.6 | 0.00004 |
| ILMN_1371503 | RGD1561694_predicted | 2.3 | 0.01136 | 6.2 | 0.00002 |
| ILMN_1356153 | RGD1561749_predicted | 2.0 | 0.0167 | 7.9 | 0.00002 |
| ILMN_1365004 | RGD1562047_predicted | 3.6 | 0.00726 | 18.7 | 0.00002 |
| ILMN_1353269 | RGD1562690_predicted | 2.3 | 0.00553 | 3.5 | 0.00004 |
| ILMN_1374906 | RGD1562758_predicted | 2.5 | 0.00553 | 6.3 | 0.00002 |
| ILMN_1353937 | RGD1562868_predicted | 2.5 | 0.00726 | 15.5 | 0.00002 |
| ILMN_1370343 | RGD1563601_predicted | 2.3 | 0.00823 | 4.7 | 0.00002 |
| ILMN_1356239 | RGD1564216_predicted | 2.0 | 0.03348 | 5.9 | 0.00002 |
| ILMN_1367571 | RGD1564688_predicted | 2.3 | 0.0204 | 4.4 | 0.00002 |
| ILMN_1360379 | RGD1565137_predicted | 2.6 | 0.00205 | 6.3 | 0.00002 |
| ILMN_1365610 | RGD1565319_predicted | 2.2 | 0.01854 | 11.8 | 0.00002 |
| ILMN_1650736 | RGD1565368_predicted | 2.2 | 0.02494 | 3.2 | 0.00019 |
| ILMN_1365849 | RGD1565561_predicted | 2.3 | 0.01516 | 10.1 | 0.00002 |
| ILMN_1363374 | RGD1566189_predicted | 3.4 | 0.01136 | 2.8 | 0.00029 |
| ILMN_1356835 | Rgs10 | 2.5 | 0.00634 | 3.3 | 0.00002 |
| ILMN_1374391 | Rhoc_predicted | 2.0 | 0.04051 | 2.7 | 0.00002 |
| ILMN_1354552 | Rnf125_predicted | 2.1 | 0.0227 | -1.4 | 0.00019 |
| ILMN_1376935 | RT1-Da | 3.2 | 0.01021 | 2.4 | 0.00002 |
| ILMN_1365192 | S100a4 | 5.2 | 0.00292 | 14.4 | 0.00002 |
| ILMN_1350690 | S100a8 | 2.5 | 0.01516 | 2.0 | 0.00063 |
| ILMN_1356076 | Sart2_predicted | 2.8 | 0.00089 | 3.9 | 0.00002 |
| ILMN_1371122 | Scpep1 | 3.3 | 0.0041 | 2.1 | 0.00002 |
| ILMN_1376484 | Sept9 | 2.1 | 0.00089 | 2.6 | 0.00002 |
| ILMN_1372782 | Serpina3n | 7.6 | 0.00483 | 1.0 | 0.47123 |
| ILMN_2040557 | Serpine1 | 2.3 | 0.01021 | 2.2 | 0.00002 |
| ILMN_1355191 | Slc16a1 | 2.5 | 0.00175 | 7.1 | 0.00002 |
| ILMN_1350798 | Slc16a3 | 2.2 | 0.04885 | 3.6 | 0.00019 |
| ILMN_1365597 | Slc1a5 | 2.5 | 0.0167 | 3.6 | 0.00002 |
| ILMN_1368133 | Slc28a2 | 2.6 | 0.00823 | 1.2 | 0.15145 |
| ILMN_1358568 | Slc7a7 | 2.9 | 0.00292 | 2.7 | 0.00002 |
| ILMN_1361588 | Slfn3 | 4.8 | 0.00345 | 4.7 | 0.00004 |
| ILMN_1374499 | Snai1 | 2.0 | 0.04467 | 3.8 | 0.00002 |
| ILMN_1367263 | Sod2 | 2.0 | 0.01373 | 1.3 | 0.00044 |
| ILMN_1356384 | Sparc | 3.0 | 0.00345 | 1.6 | 0.00002 |
| ILMN_1364291 | Spbc24_predicted | 2.8 | 0.01136 | 8.9 | 0.00002 |
| ILMN_1365457 | Spbc25 | 2.0 | 0.00726 | 5.6 | 0.00002 |
| ILMN_1361495 | Spn | 2.6 | 0.01136 | 14.6 | 0.00002 |
| ILMN_1352642 | Spp1 | 4.3 | 0.00553 | 18.3 | 0.00002 |
| ILMN_1361562 | St3gal4 | 2.1 | 0.01136 | 2.1 | 0.00002 |
| ILMN_1357673 | Stab1_predicted | 2.3 | 0.00133 | 1.3 | 0.00007 |
| ILMN_1349306 | Steap1_predicted | 3.3 | 0.00244 | 12.6 | 0.00002 |
| ILMN_1349419 | Stmn1 | 2.6 | 0.00553 | 9.0 | 0.00002 |
| ILMN_1348927 | Tbxas1 | 3.0 | 0.00244 | 3.6 | 0.00002 |
| ILMN_1352889 | Tctex1 | 2.3 | 0.02748 | 4.2 | 0.00002 |
| ILMN_1376249 | Tf | 4.1 | 0.00244 | -1.4 | 0.04965 |
| ILMN_1356448 | Tgfb1 | 2.4 | 0.00823 | 3.7 | 0.00002 |
| ILMN_1371070 | Tgfbi | 2.2 | 0.00205 | 4.9 | 0.00002 |
| ILMN_1360569 | Th | 5.4 | 0.01021 | 35.0 | 0.00002 |
| ILMN_1650153 | Thbs2 | 2.4 | 0.00634 | 4.0 | 0.00011 |
| ILMN_1353896 | Tlr2 | 3.4 | 0.00553 | 1.6 | 0.00029 |
| ILMN_1650719 | Tmem106a | 2.0 | 0.04051 | 2.5 | 0.00002 |
| ILMN_1364908 | Tmepai_predicted | 3.1 | 0.02494 | 4.0 | 0.00002 |
| ILMN_1357580 | Tnfaip8l2 | 2.0 | 0.00345 | 1.3 | 0.00002 |
| ILMN_1373313 | Tnfrsf1a | 2.4 | 0.00292 | 1.6 | 0.00002 |
| ILMN_1348834 | Tnfsf13 | 2.2 | 0.00634 | 1.2 | 0.0108 |
| ILMN_1368083 | Tpbg | 2.2 | 0.04885 | 12.0 | 0.00002 |
| ILMN_1650818 | Tpi1 | 2.2 | 0.01244 | 3.8 | 0.00011 |
| ILMN_1649963 | Tpm3 | 2.0 | 0.00553 | 2.8 | 0.00002 |
| ILMN_1362370 | Tpst2 | 2.3 | 0.00553 | 5.4 | 0.00002 |
| ILMN_1355984 | Tpx2_predicted | 2.6 | 0.00553 | 6.8 | 0.00002 |
| ILMN_1371160 | Traf4af1 | 2.4 | 0.01021 | 8.6 | 0.00002 |
| ILMN_1357948 | Trem1_predicted | 2.6 | 0.00345 | 1.8 | 0.00002 |
| ILMN_1348883 | Trem2_predicted | 3.9 | 0.01021 | 2.1 | 0.00044 |
| ILMN_1354206 | Tuba1 | 2.5 | 0.00634 | 4.1 | 0.00002 |
| ILMN_1370544 | Tubb5 | 2.9 | 0.00175 | 5.4 | 0.00002 |
| ILMN_1358531 | Tubb6 | 3.1 | 0.00553 | 5.3 | 0.00002 |
| ILMN_1372404 | Tyrobp | 3.3 | 0.00292 | 2.7 | 0.00002 |
| ILMN_1371910 | Ube2e3_predicted | 2.0 | 0.03049 | 3.9 | 0.00002 |
| ILMN_1370862 | Upp1 | 3.9 | 0.01021 | 6.3 | 0.00002 |
| ILMN_1364472 | Vcam1 | 2.9 | 0.0008 | 2.3 | 0.00002 |
| ILMN_1364377 | Vim | 2.9 | 0.00823 | 6.1 | 0.00002 |
| ILMN_1370122 | Was_predicted | 2.0 | 0.0041 | 1.6 | 0.00002 |
| ILMN_1362680 | Waspip | 2.5 | 0.00147 | 2.7 | 0.00002 |
| **Genes upregulated in TINT** | | | | | |
| **Probe_ID** | **Symbol** | **Fold change TINT vs. control** | ***P*-value** | **Fold change tumor vs. control** | ***P*-value** |
| ILMN_1352975 | A2m | 4.0 | 0.01854 | 1.6 | 0.00063 |
| ILMN_1362628 | Abcg2 | 3.5 | 0.01244 | 3.2 | 0.00002 |
| ILMN_1358403 | Adcy4 | 2.3 | 0.0167 | 1.3 | 0.00029 |
| ILMN_1357059 | Alox5ap | 3.0 | 0.00726 | 1.3 | 0.00409 |
| ILMN_1376877 | Arhgap9 | 2.0 | 0.02748 | -1.4 | 0.0203 |
| ILMN_1365368 | Basp1 | 2.4 | 0.0227 | 1.3 | 0.07843 |
| ILMN_1352142 | C1qb | 6.1 | 0.00345 | 2.8 | 0.00044 |
| ILMN_1359739 | C1qg | 4.4 | 0.00919 | 1.9 | 0.00091 |
| ILMN_1350943 | Cd163_predicted | 6.0 | 0.00069 | 1.4 | 0.01659 |
| ILMN_1361192 | Cd48 | 2.1 | 0.04051 | 1.4 | 0.00174 |
| ILMN_1366566 | Cd68 | 4.9 | 0.00726 | 1.8 | 0.0068 |
| ILMN_1360190 | Cd8a | 2.1 | 0.00133 | 1.1 | 0.54199 |
| ILMN_1354755 | Centa2 | 2.0 | 0.01854 | 1.0 | 0.68825 |
| ILMN_1351404 | Cfb | 3.2 | 0.00133 | -3.3 | 0.00091 |
| ILMN_1369323 | Chp2 | 2.0 | 0.00634 | -1.1 | 0.0068 |
| ILMN_1362842 | Cilp_predicted | 4.7 | 0.00292 | 1.0 | 0.98303 |
| ILMN_1365425 | Cma1 | 3.4 | 0.01244 | -3.3 | 0.00004 |
| ILMN_1376891 | Cndp2 | 2.3 | 0.0167 | -1.7 | 0.00019 |
| ILMN_1374380 | Col14a1_predicted | 3.1 | 0.00345 | 1.0 | 0.9484 |
| ILMN_1365619 | Cst7_predicted | 2.1 | 0.02494 | 1.1 | 0.00862 |
| ILMN_1361478 | Cthrc1 | 5.3 | 0.01373 | 2.9 | 0.00002 |
| ILMN_1359951 | Ctsc | 2.3 | 0.0167 | 1.6 | 0.00126 |
| ILMN_1367864 | Ctsh | 2.1 | 0.01516 | -1.4 | 0.00019 |
| ILMN_1369599 | Cxcl4 | 5.1 | 0.00175 | 1.1 | 0.11838 |
| ILMN_1352424 | Cybb | 2.0 | 0.0167 | 1.5 | 0.00044 |
| ILMN_1372919 | Cyr61 | 2.2 | 0.01021 | 1.4 | 0.09047 |
| ILMN_1376838 | Dcir3 | 4.1 | 0.01373 | 2.2 | 0.00004 |
| ILMN_1349412 | Dock11 | 2.5 | 0.00147 | 1.4 | 0.00091 |
| ILMN_1370366 | Dpp7 | 3.1 | 0.00089 | 1.0 | 0.76405 |
| ILMN_1358233 | Eln | 3.4 | 0.00205 | -1.3 | 0.00126 |
| ILMN_1376301 | Enpp3 | 3.2 | 0.00553 | 1.7 | 0.00007 |
| ILMN_1351676 | Esam | 2.4 | 0.02494 | 1.5 | 0.00002 |
| ILMN_1352360 | F13a1 | 2.0 | 0.0041 | 1.0 | 0.83979 |
| ILMN_1356793 | F3 | 2.4 | 0.0167 | 1.3 | 0.00234 |
| ILMN_1371753 | F5_mapped | 2.0 | 0.01244 | 1.1 | 0.23592 |
| ILMN_1351076 | Fbn2 | 2.3 | 0.00345 | 1.2 | 0.00862 |
| ILMN_1350117 | Fcgrt | 4.0 | 0.00345 | -1.1 | 0.34327 |
| ILMN_1349211 | Fcna | 4.4 | 0.00244 | -2.0 | 0.00007 |
| ILMN_1354017 | Folr2_predicted | 4.0 | 0.01373 | 1.2 | 0.00862 |
| ILMN_1375247 | Fxyd2 | 2.6 | 0.01373 | -1.3 | 0.01659 |
| ILMN_1357452 | Gda | 3.6 | 0.0008 | 1.0 | 0.87634 |
| ILMN_1349855 | Gm2a | 2.4 | 0.03681 | 1.2 | 0.05809 |
| ILMN_1376482 | Hcls1 | 2.3 | 0.00147 | 1.3 | 0.00002 |
| ILMN_1650285 | Hmox1 | 14.5 | 0.00133 | 2.3 | 0.00091 |
| ILMN_1370611 | Hpse | 2.3 | 0.00147 | 1.4 | 0.00091 |
| ILMN_1370795 | Ifitm1_predicted | 3.1 | 0.01136 | 1.7 | 0.00126 |
| ILMN_1352762 | Ifitm3 | 2.0 | 0.02748 | -1.3 | 0.05809 |
| ILMN_1349716 | Igsf7 | 3.9 | 0.0227 | 1.3 | 0.00531 |
| ILMN_1375567 | Kng1 | 3.3 | 0.0204 | 1.0 | 0.01659 |
| ILMN_1357030 | Laptm5 | 4.3 | 0.01136 | 1.8 | 0.00063 |
| ILMN_1376661 | Lbp | 4.2 | 0.00726 | -1.1 | 0.21249 |
| ILMN_1363606 | Lcn2 | 2.3 | 0.03348 | -2.0 | 0.23592 |
| ILMN_1370061 | LOC310877 | 2.2 | 0.0167 | -1.3 | 0.02462 |
| ILMN_1354702 | LOC362934 | 2.1 | 0.04467 | -1.7 | 0.00862 |
| ILMN_1361251 | LOC497811 | 2.7 | 0.00175 | -1.7 | 0.00007 |
| ILMN_1351139 | LOC498279 | 5.0 | 0.00553 | 2.5 | 0.00029 |
| ILMN_1372199 | LOC501744 | 3.9 | 0.01136 | 2.1 | 0.00091 |
| ILMN_1366909 | LOC683463 | 3.0 | 0.00919 | 1.2 | 0.00234 |
| ILMN_1356957 | LOC687856 | 2.3 | 0.02494 | 1.9 | 0.00002 |
| ILMN_1353683 | LOC688750 | 2.0 | 0.00175 | -1.3 | 0.00002 |
| ILMN_1376823 | Lox | 5.2 | 0.0008 | 1.5 | 0.04965 |
| ILMN_1376846 | Loxl1 | 3.7 | 0.00175 | 1.7 | 0.00004 |
| ILMN_1355909 | Lr8 | 2.8 | 0.03348 | 1.5 | 0.00063 |
| ILMN_1356330 | Lst1 | 2.1 | 0.01136 | 1.6 | 0.00002 |
| ILMN_1352701 | Lum | 2.0 | 0.01136 | -1.1 | 0.47123 |
| ILMN_1374969 | Lyl1 | 3.7 | 0.00919 | 1.4 | 0.00174 |
| ILMN_1375563 | Maf | 2.5 | 0.00634 | -1.25 | 0.11838 |
| ILMN_1371205 | Map4k1_predicted | 2.5 | 0.01136 | 1.4 | 0.00174 |
| ILMN_1355362 | Mca32 | 2.3 | 0.00175 | 1.3 | 0.00091 |
| ILMN_1371491 | Metrnl | 2.7 | 0.01021 | 1.4 | 0.0108 |
| ILMN_1365298 | Mfap5_predicted | 2.4 | 0.02494 | -2.0 | 0.00002 |
| ILMN_1351917 | Mgp | 3.5 | 0.00345 | 1.3 | 0.26084 |
| ILMN_1374846 | Mrc1_predicted | 5.6 | 0.00205 | 2.0 | 0.00063 |
| ILMN_1363346 | Ms4a6a_predicted | 3.4 | 0.00634 | 2.5 | 0.00007 |
| ILMN_1372165 | Nckap1l_predicted | 3.5 | 0.00823 | 1.4 | 0.0068 |
| ILMN_1364922 | Nid1 | 2.4 | 0.00292 | -1.4 | 0.00019 |
| ILMN_1367443 | Pdpn | 3.1 | 0.00205 | -2.0 | 0.00019 |
| ILMN_1359441 | Pla2g2a | 4.1 | 0.01021 | -1.7 | 0.07843 |
| ILMN_1358127 | Plat | 2.7 | 0.00292 | 1.3 | 0.00862 |
| ILMN_1376575 | Pltp_predicted | 2.5 | 0.01244 | -1.7 | 0.00019 |
| ILMN_1370798 | Plxdc2_predicted | 2.0 | 0.02748 | -2.0 | 0.00002 |
| ILMN_1373876 | Pmp22 | 2.1 | 0.00919 | -3.3 | 0.00002 |
| ILMN_1361790 | Ptgis | 2.5 | 0.03049 | -3.3 | 0.00002 |
| ILMN_1360759 | Ptpns1 | 2.3 | 0.00292 | 1.2 | 0.07843 |
| ILMN_1349137 | Ptpro | 3.0 | 0.0167 | 1.2 | 0.04207 |
| ILMN_1365800 | Rac2 | 3.8 | 0.00726 | 1.8 | 0.00063 |
| ILMN_1651173 | Rarres1 | 2.8 | 0.0167 | 1.0 | 0.3731 |
| ILMN_1371681 | Rcn3_predicted | 2.3 | 0.01373 | -1.4 | 0.00063 |
| ILMN_1364607 | RGD1308734 | 2.2 | 0.01244 | 1.4 | 0.00313 |
| ILMN_1650931 | RGD1560062_predicted | 2.1 | 0.0227 | 1.0 | 0.72586 |
| ILMN_1349268 | Rhoa | 2.4 | 0.0227 | -2.0 | 0.54199 |
| ILMN_1376459 | RT1-Db1 | 3.0 | 0.03681 | 1.7 | 0.00234 |
| ILMN_1368826 | Rtp4_predicted | 2.2 | 0.0204 | -1.1 | 0.9484 |
| ILMN_1350770 | Scn3b | 3.0 | 0.00345 | 1.1 | 0.21249 |
| ILMN_1371209 | Sectm1 | 3.8 | 0.01516 | -1.4 | 0.00044 |
| ILMN_1352285 | Serpinb1a | 2.0 | 0.01854 | -1.1 | 0.83979 |
| ILMN_1376417 | Serpine1 | 2.7 | 0.04467 | 2.1 | 0.00002 |
| ILMN_1374314 | Serping1 | 2.5 | 0.01021 | -5.0 | 0.00002 |
| ILMN_1369218 | Sfrp2 | 6.8 | 0.00069 | -1.4 | 0.00862 |
| ILMN_1375152 | Sh3bgrl3_predicted | 2.5 | 0.01373 | 1.4 | 0.0108 |
| ILMN_1360795 | Slc11a1 | 2.2 | 0.01854 | 1.1 | 0.00531 |
| ILMN_1351485 | Slc40a1 | 3.1 | 0.00205 | 1.3 | 0.01659 |
| ILMN_1357807 | Slit3 | 2.6 | 0.00175 | -1.1 | 0.01347 |
| ILMN_1360286 | Slpi | 6.0 | 0.00726 | 2.9 | 0.00174 |
| ILMN_1650334 | Spon1 | 2.4 | 0.00147 | -2.5 | 0.00002 |
| ILMN_1373216 | Srpx | 3.6 | 0.00069 | 1.0 | 0.83979 |
| ILMN_1362820 | Ssg1 | 3.5 | 0.00147 | -1.1 | 0.68825 |
| ILMN_1366184 | Sulf2 | 2.9 | 0.00292 | 1.6 | 0.00063 |
| ILMN_1350042 | Thbs4 | 4.5 | 0.03049 | 1.0 | 0.72586 |
| ILMN_1359200 | Timp1 | 3.3 | 0.0227 | 2.5 | 0.00002 |
| ILMN_1374220 | Tnfrsf11b | 2.6 | 0.00244 | -2.0 | 0.00091 |
| ILMN_1349103 | Tpsab1 | 2.5 | 0.01021 | -1.4 | 0.0068 |
| ILMN_1360233 | Trpv4 | 2.1 | 0.00634 | -1.4 | 0.00002 |
| ILMN_1353991 | Unc93b1 | 3.1 | 0.01136 | 1.4 | 0.00126 |
| ILMN_1348914 | Vav1 | 2.0 | 0.03049 | 1.3 | 0.00091 |
| ILMN_1363923 | Xlkd1_predicted | 5.2 | 0.00069 | 1.0 | 0.91319 |
